# The Intimate Connection Between Lipids and Hedgehog Signaling

**DOI:** 10.3389/fcell.2022.876815

**Published:** 2022-06-09

**Authors:** Thi D. Nguyen, Melissa E. Truong, Jeremy F. Reiter

**Affiliations:** ^1^ Department of Biochemistry and Biophysics, Cardiovascular Research Institute, University of California, San Francisco, San Francisco, CA, United States; ^2^ Division of Biology and Biological Engineering, California Institute of Technology, Pasadena, CA, United States; ^3^ Chan Zuckerberg Biohub, San Francisco, CA, United States

**Keywords:** intercellular signaling, development, cholesterolyation, sterols, cilia

## Abstract

Hedgehog (HH) signaling is an intercellular communication pathway involved in directing the development and homeostasis of metazoans. HH signaling depends on lipids that covalently modify HH proteins and participate in signal transduction downstream. In many animals, the HH pathway requires the primary cilium, an organelle with a specialized protein and lipid composition. Here, we review the intimate connection between HH signaling and lipids. We highlight how lipids in the primary cilium can create a specialized microenvironment to facilitate signaling, and how HH and components of the HH signal transduction pathway use lipids to communicate between cells.

## Introduction

The HH pathway functions in metazoan development as one of the principal means of cell-cell communication (Ingham and McMahon, 2001; Ingham, 2018). HH was discovered in a *Drosophila* genetic screen for developmental regulators (Nüsslein-Volhard and Wieschaus, 1980). HH proteins are secreted ligands that are interpreted by receiving cells via the transmembrane proteins Patched (PTCH) and Smoothened (SMO) to control the activity of the downstream transcription factor effectors, called Cubitus interruptus in *Drosophila* and GLI in vertebrates (Nüsslein-Volhard and Wieschaus, 1980; Nüsslein-Volhard et al., 1984; Forbes et al., 1993; Quirk et al., 1997).

HH signaling is one fundamental mechanism by which cells communicate and is deployed both in development and adult physiology to control diverse tissue dynamics, including patterning and the regulation of cell growth. Consequently, defective HH signaling in development causes birth defects, and mis-activation of HH signaling postnatally can cause cancer.

As many HH pathway components are conserved between insects and vertebrates, it was unexpected when a genetic screen in mice identified proteins required for both vertebrate HH signaling and the formation of an organelle called the primary cilium (Huangfu et al., 2003). The primary cilium is a microtubule-based organelle found on most vertebrate cells (Wheatley, 1995; Wheatley et al., 1996). Unlike motile cilia, such as those found on cells in the airway, the brain ventricles, and the oviduct that beat to move overlying fluid, primary cilia are immotile and specialized for signal transduction (Ishikawa and Marshall, 2011).

**FIGURE 1:**
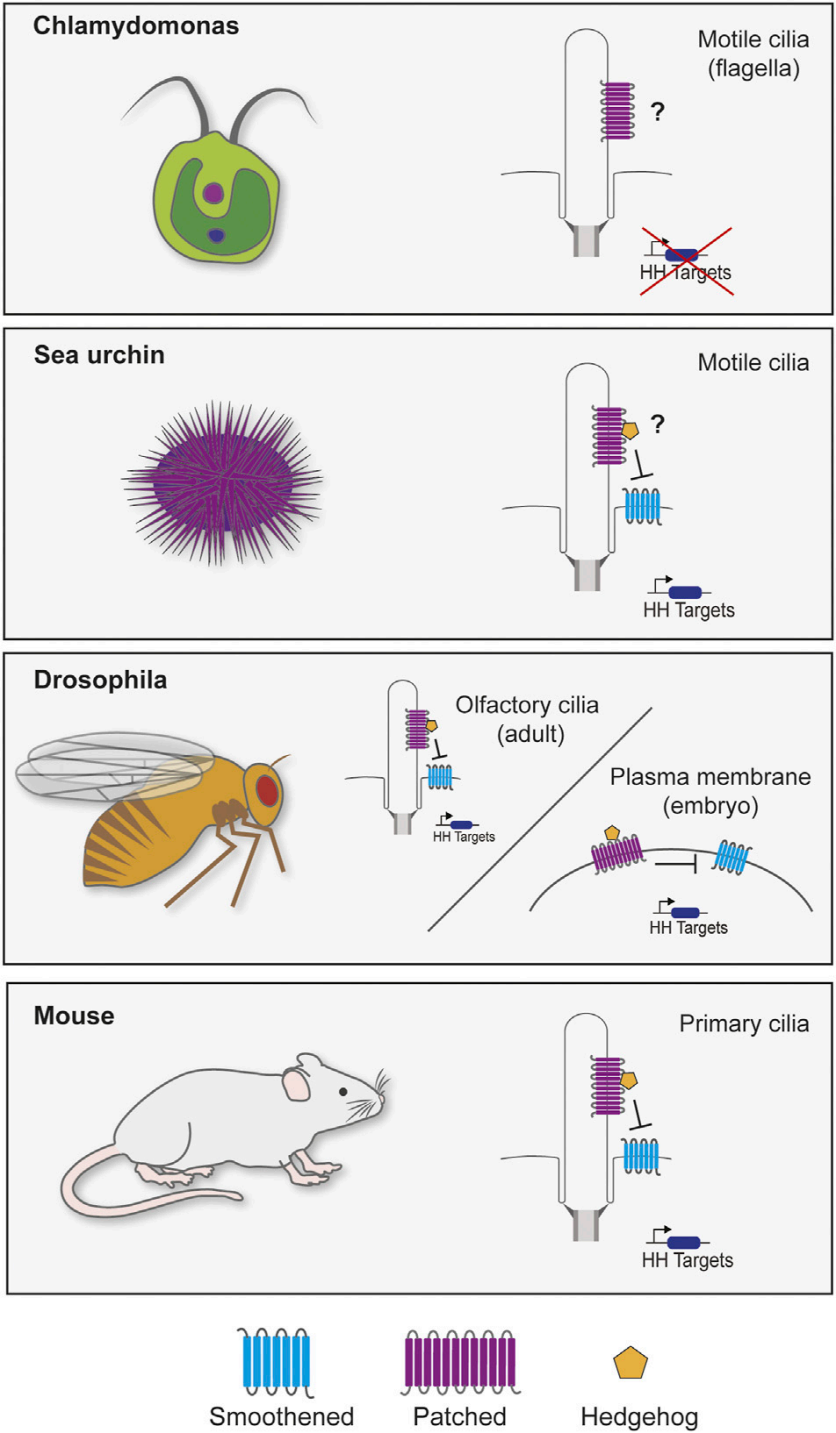
Cilia and Hedgehog signaling throughout evolution. Though cilia are highly conserved, the reliance of HH signaling on the cilium varies through evolution. The green alga *Chlamydomonas reinhardtii* genome possesses two homologs of *PTCH* (Cre02.g093500 and Cre12.g496350), but not other components of the HH pathway. It will be interesting to determine whether either acts at the *Chlamydomonas* flagella. In sea urchin embryos, SMO localizes to cilia to activate HH signaling for mesoderm specification (Warner et al., 2014). Different tissues in *Drosophila* transduce HH signals with or without cilia. Most *Drosophila* cells lack cilia and transduce HH signals via Smo at the plasma membrane (Zhu et al., 2003; Jia et al., 2004). However, olfactory sensory neuron cilia in the adult fly brain signal through ciliary Smo (Kuzhandaivel et al., 2014). Vertebrates require primary cilia to transduce HH signals (Bangs and Anderson, 2017). Defects in ciliary transport or structure cause a wide range of HH-related phenotypes (Reiter and Leroux, 2017).

The discovery that primary cilia are required to transduce mammalian HH signaling sparked investigation into the connection between HH signaling and the primary cilium (Bangs and Anderson, 2017). Research into primary cilia in diverse organisms has revealed that evolution has played with the role of cilia in transducing HH signals. Cilia are present in all clades of extant eukaryotes, indicating that they were probably present in the last eukaryotic common ancestor (LECA), whereas the HH pathway probably arose with multicellularity (Figure 1).

In vertebrates, coordinated protein trafficking of HH pathway components into and out of cilia is required for regulated signal transduction. In the absence of HH signals, Patched1 (PTCH1) and the G protein-coupled receptor GPR161 localize to the ciliary membrane (Rohatgi et al., 2007; Mukhopadhyay et al., 2013). Binding of a HH ligand, such as Sonic Hedgehog (SHH), to PTCH1 triggers exit of PTCH1 from the cilium which cues ciliary accumulation of SMO (Corbit et al., 2005). Once localized to the cilium, SMO converts GLI proteins, which localize to the ciliary tip, into transcriptional activators which leave the cilium, enter the nucleus, and induce HH target genes (Haycraft et al., 2005; Wen et al., 2010; Santos and Reiter, 2014).

One theoretical evolutionary advantage of scaffolding signal transduction within the primary cilium is that it may increase signaling fidelity by imposing an additional level of regulation through subcellular trafficking. Although the primary cilium shares a membrane that is contiguous with the plasma membrane, the cilium can signal distinctly from the rest of the cell (Delling et al., 2013; Marley et al., 2013; Truong et al., 2021). Key to its signaling functions is the maintenance of distinct ciliary protein and lipid compositions (Nachury, 2014; Mick et al., 2015).

Over the last decade, we have gained some understanding of how the protein composition of the cilium is controlled. For example, a region near the base of the cilium called the transition zone, recognized electron micrographically by prominent structures called Y-fibers connecting the axoneme to the ciliary membrane, controls protein accumulation within the cilium (Garcia-Gonzalo and Reiter, 2017; Nachury and Mick, 2019).

The distinct protein composition of the ciliary membrane raises the interesting question of whether the lipid composition of the ciliary membrane similarly differs from that of other cellular membranes. Less is understood about how different lipids are distributed throughout the cell, including at the cilium.

Broadly speaking, lipids play three biological functions: as energy storage, as the principal components of cellular membranes, and as participants in signal transduction. Lipid droplets store neutral lipids that can be catabolized to generate ATP. Cellular membranes are primarily composed of bilayers of amphipathic phospholipids. Other lipids, such as sterols and phosphoinositides, are non-uniformly distributed and define distinct cellular membranes. Subcellular differences in lipid composition affect membrane curvature, tension, and the function of signaling proteins (van Meer et al., 2008; Harayama and Riezman, 2018).

One intercellular communication pathway dependent on lipids is HH signaling. For example, lipidation of HH ligands is key to their activity and extracellular distribution as gradients to pattern developing tissues (Eaton, 2008). Downstream of HH, the HH receptor PTCH1 transports sterols to affect the composition of the membrane (Zhang et al., 2018; Kinnebrew et al., 2021; Qi et al., 2019). Sterols also regulate the activity of the central HH pathway component SMO (Cooper et al., 2003; Myers et al., 2013, 2017; Nachtergaele et al., 2013; Nedelcu et al., 2013; Byrne et al., 2016; Huang et al., 2016; Luchetti et al., 2016; Xiao et al., 2017; Huang et al., 2018; Raleigh et al., 2018). Still other lipids, phosphoinositides, are read out by TUBBY family proteins to control the trafficking of HH signal transduction component GPR161 to cilia (Chávez et al., 2015; Garcia-Gonzalo et al., 2015). In this review, we focus on the role of lipids in HH signaling, especially at the ciliary membrane. We examine how the lipid composition of the primary cilium creates a specialized microenvironment essential for vertebrate HH signaling. Additionally, we dissect how these lipids function in embryonic development and how their dysregulation causes birth defects. Further research into how lipids function in HH signaling, particularly within the primary cilium, may illuminate general principles by which the subcellular distribution of lipids is controlled to contribute to protein function and the propagation of information.

## Ciliary Membranes Have a Distinct Lipid Composition

In protists, biochemical assessments have indicated that the lipid composition of cilia is distinct. For example, the ciliary membranes of *Paramecia* and *Tetrahymena* are enriched in phosphonolipids (consisting of the well-named ciliatine attached to a lipid backbone) and sphingolipids (Kennedy and Thompson, 1970; Smith et al., 1970; Andrews and Nelson, 1979; Kaneshiro et al., 1984). In *Paramecia*, a mutation that alters ciliary sphingolipid levels compromises the function of voltage-sensitive channels, suggesting that its distinct lipid composition is critical for ciliary protein function and that sphingolipids may be particularly important for ciliary biology (Forte et al., 1981).

One sphingolipid, sphingomyelin, can sequester sterols in complexes (Leathes, 1925; McConnell and Radhakrishnan, 2003; Das et al., 2014). Filipin, a mixture of polyene macrolides, binds 3-β-hydroxysterols and can be observed in freeze-fracture electron microscopy (Kinsky et al., 1966). In the distantly related protists Euglena and Trypanosomes, filipin staining revealed that sterols are enriched in the flagellar membrane (Melkonian et al., 1982; Souto-Padrón and de Souza, 1986; Tetley, 1986). In quail, filipin staining also demonstrated robust enrichment of 3-β-hydroxysterols in the ciliary membrane (Chailley and Boisvieux-Ulrich, 1985). Similarly, Laurdan staining of ordered lipids suggested that ciliary membranes are enriched in sterols (Tyler et al., 2009). As described further below, sterols contribute to HH signaling, and thus ciliary sphingolipids, by controlling the accessibility of sterols, can limit the signaling functions of the cilium. Indeed, sphingomyelin biosynthetic pathway enzymes restrain HH signaling (Kinnebrew et al., 2019).

How else might ciliary lipids contribute to ciliary protein function? One possibility is that they function as specific cofactors for ciliary proteins. Some lipids, such as phosphoinositides, may be at lower molar concentrations than their interacting proteins and thus may function as regulatory cofactors. Another possibility is that ciliary lipids impart a distinct biophysical or biochemical property to the ciliary membrane which is itself important for protein function. Lipids help determine membrane viscosity, surface charge and ion-binding capacity. By affecting any of these parameters, ciliary lipids may affect signal transduction by ciliary proteins, and perhaps especially ciliary membrane-associated proteins.

The ciliary membrane consists of a fraction of the cellular membrane, less than 0.01% of the total (Mukhopadhyay et al., 2017) and, to date, lipidomic characterizations of cilia have been restricted to those of organisms from which cilia can be collected in biochemical quantities (Lobasso et al., 2010; Raleigh et al., 2018). Previously, we fractionated membranes of sea urchin cilia from other cellular membranes and discovered that sea urchin cilia were enriched in several oxysterols, oxygenated derivatives of cholesterol (Raleigh et al., 2018).

Due to technical challenges in purifying mammalian primary ciliary membranes, we know less about which lipids compose vertebrate primary cilia than the cilia of protists and invertebrates. Techniques for determining the subcellular localization of lipids lag behind equivalent approaches for proteins. For example, proximity labeling approaches have greatly accelerated elucidation of the mammalian ciliary proteome (Mick et al., 2015). The ability to label lipids in specific subcellular domains does not currently exist, but its development would be a boon to comparing the lipid composition of many subcellular membranes, not just that of the ciliary membrane. Similarly, fluorescence imaging of lipids is hampered by the lack of molecular probes for most lipids (Balla and Várnai, 2002; Wills et al., 2018).

Because of the limitations to identifying ciliary lipids in vertebrate cells, we do not know whether the enrichment of sphingolipids and sterols extends to the many types of animal cilia. Indeed, staining of mammalian cilia for sterols has shown conflicting results about whether sterols are enriched (Nelson et al., 2008; Breslow et al., 2013; Kinnebrew et al., 2019; Miyamoto et al., 2020). Thus, sterol enrichment in cilia may be cell type-specific or be limited to a class of sterols detected by specific visualization methods.

However, like sea urchin, sea anemone, and protists, mammalian sperm can be fractionated into their heads, analogous to cell bodies, and tails, analogous to cilia (Toshimori et al., 1985; Connor et al., 1998; Mourvaki et al., 2010). Sterol levels in the sperm heads and tails differ, suggesting that, as in protists, lipids may be differentially distributed between the cilium and other subcellular compartments in animal cells.

## Ciliary Phosphoinositides Regulate GPCR Delivery and HH Signaling

Recent reviews have described how lipids contribute to ciliary structure (Garcia et al., 2018; Nechipurenko, 2020). In this section, we focus specifically on how ciliary lipids participate in the transduction of HH signals, the best understood of the intercellular cues communicated via cilia. The best understood of the lipids participating in ciliary signaling are the phosphoinositides.

Phosphoinositides are phosphorylated lipids that confer molecular identity to cellular membranes (di Paolo and de Camilli, 2006; Shewan et al., 2011). Reversible phosphorylation of phosphatidylinositol can give rise to seven distinct phosphoinositides which exhibit distinct subcellular distributions (Schink et al., 2016). For instance, the Golgi membrane is enriched in PI(4)P, whereas the nuclear envelope is enriched in PI(5)P (Shewan et al., 2011). Physical separation of these membranes helps partition these distinct phosphoinositides.

**FIGURE 2:**
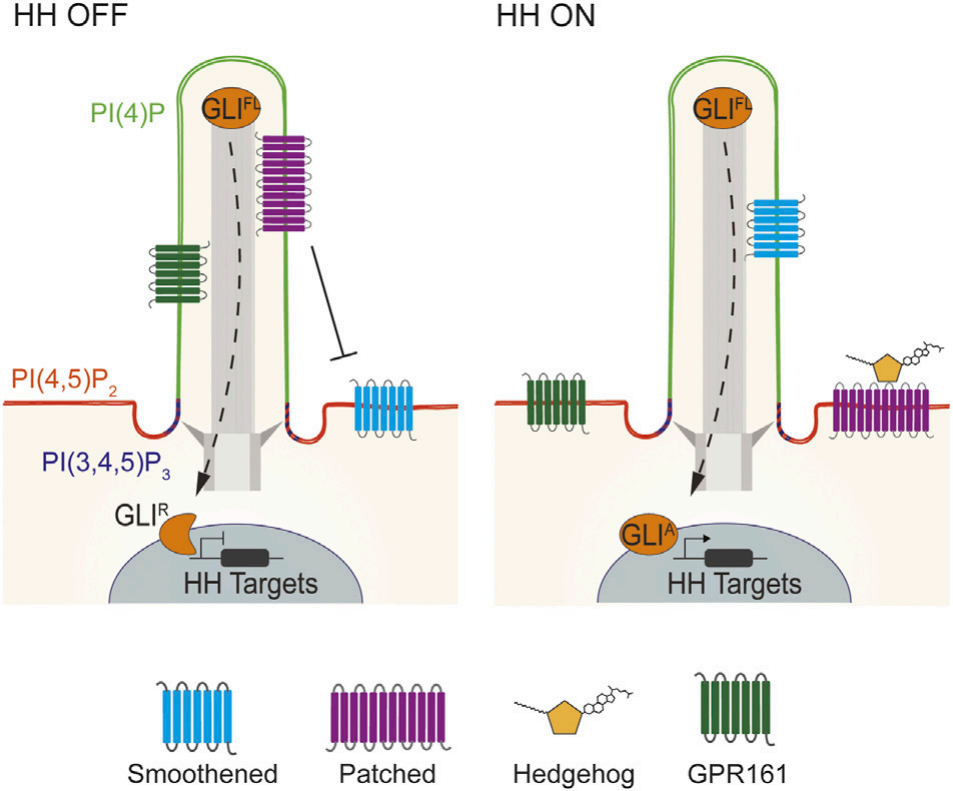
Lipid domains within vertebrate cilia. Vertebrate HH signaling depends on the tightly coordinated trafficking of pathway components into and out of cilia. In the absence of HH signals, PTCH1 localizes to cilia and represses SMO. Consequently, GLI transcription factors are proteolytically processed to their repressor state to inhibit HH target genes. HH binding to PTCH1 leads to SMO accumulation in cilia, GPR161 exit, and GLI activator formation to induce HH target gene expression (Bangs and Anderson, 2017; Kong et al., 2019). Specialized membrane domains within mouse and human cilia allow for HH signal transduction. Mouse and human cilia are enriched in the phosphoinositide PI(4)P, whereas PI(4,5)P_2_ is enriched outside of the cilium. Defects in the distribution of these two lipids causes mislocalization of HH pathway components such as GPR161 to cause birth defects (Chávez et al., 2015; Garcia-Gonzalo et al., 2015). In contrast to PI(4)P, PI(4,5)P_2_ and PI(3,4,5)P_3_ are enriched at the transition zone at the ciliary base (Dyson et al., 2017; Conduit and Vanhaesebroeck, 2020).

Thus, it is surprising that the phosphoinositide compositions of the ciliary and plasma membranes are distinct despite being contiguous, with the ciliary membrane being relatively enriched in PI(4)P and the plasma membrane relatively enriched in PI(4,5)P_2_ (Conduit and Vanhaesebroeck, 2020; Conduit et al., 2021). An additional domain of PI(3,4,5)P_3_ localizes near the ciliary base (Figure 2) (Dyson et al., 2017).

How might this phosphoinositide boundary be maintained? One strategy for maintaining distinct lipid compositions within a contiguous membrane is through control of the localization of lipid biosynthetic enzymes. In mammals, three phosphoinositide 5-phosphatases (INPP5E, INPP5B and OCRL) convert PI(4,5)P_2_ into PI(4)P and localize to primary cilium (Bielas et al., 2009; Jacoby et al., 2009; Luo et al., 2012, 2013). Though these proteins may share overlapping functions, INPP5E is required to generate PI(4)P in the primary cilia of many cells (Chávez et al., 2015; Garcia-Gonzalo et al., 2015).

Maintenance of elevated PI(4)P and depleted PI(4,5)P_2_ within the ciliary membrane is critical for HH signal transduction (Chávez et al., 2015; Garcia-Gonzalo et al., 2015; Dyson et al., 2017). Loss of INPP5E reduces ciliary PI(4)P and increases ciliary PI(4,5)P_2_. The Tubby-family protein TULP3 binds PI(4,5)P_2_ to control the delivery of a negative regulator of HH signaling, GPR161, to cilia. In the absence of INPP5E and ciliary PI(4)P, TULP3 and GPR161 mis-accumulate in cilia (Chávez et al., 2015; Garcia-Gonzalo et al., 2015). GPR161 activates protein kinase A (PKA), a negative regulator of GLI activity via direct binding to the regulatory subunit of PKA (PKA-R), and constitutive coupling to the G-protein Gαs, to generate cAMP, the principal PKA activator (Mukhopadhyay et al., 2010, 2013; Bachmann et al., 2016). We recently identified a pool of ciliary PKA (Truong et al., 2021). Thus, mis-activation of ciliary PKA to tonically inhibit GLI activity is likely to be how loss of INPP5E or ciliary PI(4)P suppresses HH signaling. Future work may elucidate how TULP3, and its paralogs including the obesity-associated protein TUBBY, read ciliary phosphoinositide composition to limit the ciliary localization of GPR161 and perhaps other GPCRs.

Aside from affecting GPCR localization, ciliary phosphoinositides may also directly affect GPCR function. Phosphoinositides can stabilize GPCR active states or enhance specific G-protein coupling (Yen et al., 2018). Differences in phosphoinositide composition between the ciliary and plasma membranes may allow cells to control GPCR output with spatial precision. For example, perhaps ciliary GPCRs, such as GPR161, may be tuned to be active specifically in domains rich in PI(4)P. And perhaps other GPCRs, such as FFAR4, which may operate at the ciliary membrane in preadipocytes and at the plasma membrane in adipocytes, may couple differently to G-proteins in these two different domains to allow for different outputs at different stages of differentiation (Hilgendorf et al., 2019).

## Ciliary Sterols Activate Smoothened

Sterol lipids are a diverse class of lipids synthesized by the mevalonate pathway. Both cholesterol, the predominant sterol in vertebrate cells, and select oxysterols can bind to SMO to activate the HH pathway (Cooper et al., 2003; Dwyer et al., 2007; Myers et al., 2017). As SMO localization to primary cilia is required for activation of the HH pathway, the sterol composition of the ciliary membrane may contribute to SMO function. A recent study used a loss-of-function CRISPR-based approach to identify sterol biosynthetic genes that influence the strength of HH signaling (Kinnebrew et al., 2019). Liquid chromatography-tandem mass spectrometry of biochemically isolated sea urchin and porcine renal cells (LLC-PK1) helped to identify SMO-activating oxysterols enriched in cilia (Raleigh et al., 2018).

Unlike the case with phosphoinositides, there is not clear evidence of enriched localization of sterol or oxysterol catabolic enzymes at the cilium itself. However, a recent study identified sterol biosynthetic enzymes that localize at the ciliary base, including DHCR7 (Findakly et al., 2021). DHCR7 is mutated in Smith-Lemli-Opitz syndrome, an inherited disease characterized by holoprosencephaly. The holoprosencephaly is thought to be secondary to reduced HH signaling caused by the accumulation of SMO-inhibiting sterols (Fitzky et al., 1998; Wassif et al., 1998; Matsumoto et al., 2005; Nowaczyk and Irons, 2012; Sever et al., 2016). DHCR7 catalyzes the terminal step in cholesterol and 24,25-epoxycholesterol synthesis. As an integral membrane protein, DHCR7 near the ciliary base may be in the ciliary pocket membrane, a membrane invagination that surrounds the cilium (Findakly et al., 2021). DHCR7 relocalizes away from the ciliary base upon HH pathway activation, suggesting that control of the subcellular localization of sterol biosynthetic machinery may modulate ciliary lipid composition to tune HH signaling. However, understanding how sterol content in cilia is controlled remains a major challenge, particularly as existing sterol biosensors are less specific than biosensors for other lipids such as phosphoinositides (Maekawa and Fairn, 2014; Wills et al., 2018).

## Hedgehog Ligands Are Both Cholesterylated and Palmitoylated

Not only are lipids critical for creating specialized sub-cellular compartments that facilitate signaling, but lipids participate with certain core components of the HH pathway in ways critical for signaling. For example, HH proteins are covalently linked to palmitoyl and cholesterol (Porter et al., 1996a; Porter et al., 1996b; Pepinsky et al., 1998). Initially, HH is synthesized as a 45 kDa precursor comprised of a signal peptide, an N-terminal signaling domain (HhN) and a C-terminal intein (HhC) (Lee et al., 1994). Concurrent with synthesis, the signal peptide is cleaved, revealing a highly conserved N-terminal cysteine residue that is palmitoylated by Hedgehog acetyltransferase (called HHAT or SKI) (Pepinsky et al., 1998; Amanai and Jiang, 2001; Chamoun et al., 2001; Micchelli et al., 2002; Buglino and Resh, 2008). Additionally, the intein catalyzes HH cleavage and links cholesterol with the newly exposed C-terminus of HhN, thereby creating a fully processed, ∼19 kDa protein that is dually lipidated (Porter et al., 1996a; Porter et al., 1996b; Pepinsky et al., 1998). Perturbing HH lipidation has different effects in vertebrates and in *Drosophila*, which we discuss in two broad categories: signaling activity and signal distribution.

## Palmitoylation Is Important for HH Signaling Strength

The signaling potency of *Drosophila* HH and vertebrate SHH are differentially dependent on palmitoylation. In mouse fibroblast cells, non-palmitoylated SHH can still signal, albeit at reduced strength (Pepinsky et al., 1998). Similarly, non-palmitoylated SHH exhibits attenuated signaling *in vivo*, but, when overexpressed in the mouse embryonic limb bud, can, like overexpressed wild-type SHH, induce HH target genes and polydactyly (Lee et al., 2001; Chen et al., 2004).

In contrast to the mouse, un-palmitoylated Hh in *Drosophila* interferes with the signaling activity of wild-type Hh when globally overexpressed (Lee et al., 2001). Interestingly, this lack of activity seems to be specific to the ligand, and not to the system, since un-palmitoylated mouse SHH retains some signaling ability when ectopically expressed in the *Drosophila* wing disc (Chamoun et al., 2001). Un-palmitoylated HH can still partially rescue HH loss-of-function in the embryo (Gallet et al., 2003) and can induce HH signaling in the *Drosophila* wing disc (Callejo et al., 2006). Despite some species-specific dependence on palmitoylation, the palmitoyl moiety on Hedgehog proteins is critical for full signaling activity.

Cryo-EM structures of PTCH1 binding SHH reveal that SHH can bind in multiple conformations. In one conformation, the palmitoyl group makes extensive interactions in an extracellular cleft of PTCH1 composed of its two major extracellular loops, providing structural insight into one way that SHH blocks PTCH1 to activate the pathway (Qi et al., 2018a; Qi et al., 2018b; Qian et al., 2019).

## HH Cholesterylation Promotes Long-Distance Signaling

In addition to binding PTCH1 to activate the downstream pathway, the developmental functions of HH ligands in tissue patterning depend on its distribution. In the neural tube, SHH forms a gradient, highest ventrally at its sites of production, the notochord and floor plate, and decreases dorsally. In the limb bud, SHH produced posteriorly in the zone of polarizing activity decreases in concentration anteriorly. Palmitoylation of vertebrate SHH is required for long-distance signaling as un-palmitolyated SHH is largely restricted to its sites of production (Lee et al., 2001; Chen et al., 2004). Importantly, both HH and SHH proteins that lack cholesterol are still competent to induce downstream transcriptional changes in receiving cells (Porter et al., 1996a; Lewis et al., 2001; Zeng et al., 2001; Li et al., 2006). Still, un-cholesterylated SHH cannot signal over long distances (Lewis et al., 2001). Thus, both lipid modifications are critical for vertebrate HH distribution, but cholesterylation may be more relevant to the range of signaling, rather than its signaling potency.

*Drosophila* demonstrate a cell-type specific requirement for lipidation, as un-cholesterylated Hh exhibits either restricted (Porter et al., 1996b; Burke et al., 1999; Dawber et al., 2005; Callejo et al., 2006; Gallet et al., 2006; Su et al., 2007) or expanded (Gallet et al., 2003, 2006; Panáková et al., 2005) spatial distribution in different tissues.

These differences in HH distribution in different organisms or tissues represents just one way in which HH signaling can be adapted. Another difference is the requirement for primary cilia in HH signal transduction. HH signal transduction in the *Drosophila* wing disc is independent of primary cilia. Indeed, wing disc cells lack cilia. In stark contrast, vertebrate HH signal transduction requires primary cilia (Huangfu and Anderson, 2005).

Additional vertebrate-specific requirements in HH signal transduction include the involvement of Scube-family proteins, vertebrate-specific extracellular proteins that facilitate HH release from producing cells. Scube proteins, though dispensable individually, are collectively required for HH signaling (Kawakami et al., 2005; Woods and Talbot, 2005; Hollway et al., 2006; Johnson et al., 2012). *In vitro*, SCUBE2 specifically binds to and promotes the release of cholesteroylated SHH (Creanga et al., 2012; Tukachinsky et al., 2012; Wierbowski et al., 2020). Perhaps these species-specific differences in how HH signals are released from producing cells account for the different dependencies on lipidation for signaling by *Drosophila* HH and vertebrate SHH.

## HH May Communicate Over Long Distances via Multiple Mechanisms

How can HH act over multiple cell diameters as a morphogen once it is dually lipidated? As both lipid adducts on HH, cholesterol and palmitoyl, are poorly soluble in aqueous environments, HH would be expected to remain associated with membranes and not diffuse in the extracellular space. Conflicting results from studies done in *Drosophila*, zebrafish, and mouse are difficult to reconcile, raising the possibility that different organisms or different tissues distribute HH proteins in different ways. For example, there is evidence supporting the presence of HH in higher order assemblies that are less hydrophobic than monomeric lipidated HH, including as multimers, as constituents of liposomes, and as components of extracellular vesicles called exosomes.

One possibility is that HH multimerizes and internalizes its lipid moieties, exposing its hydrophilic proteinaceous face to the extracellular environment. *In vitro*, overexpressed HH will contribute to signaling-competent, high-molecular weight species in a way that depends on lipidation (Zeng et al., 2001; Chen et al., 2004; Gallet et al., 2006; Goetz et al., 2006). It remains unclear whether these high-molecular weight species exist *in vivo*.

It also is unclear whether proteins beyond HH contribute to these high-molecular weight complexes. Lipoprotein particles are extracellular macromolecular assemblies comprised of a core of esterified cholesterol moieties and triglycerides in association with apolipoproteins (Babin et al., 1999). HH can be released from *Drosophila* wing disc cells and human cultured cells as part of lipoprotein particles (Panáková et al., 2005; Palm et al., 2013). HH associated with liproprotein particles has low signaling activity (Palm et al., 2013), raising a question of whether this form of HH is critical to its function in developing tissues.

Additionally, HH may traffic on extracellular vesicles *in vitro*, in the *Drosophila* wing disc, and in developing mouse embryos (Tanaka et al., 2005; Matusek et al., 2014; Vyas et al., 2014). These extracellular vesicles may be formed via multivesicular body assembly or plasma membrane budding, mechanisms that are dependent on the endosomal sorting complex required for transport (ESCRT) (Matusek et al., 2014; Coulter et al., 2018). Whether these HH-containing extracellular vesicles have signaling capabilities and whether they can generate a morphogen gradient *in vivo* remain to be determined.

Some HH is not secreted but, rather, remains attached to the membrane and trafficked on long and thin cytonemes, specialized, actin-based cytoplasmic extensions as long as 200 µm (Kornberg, 2014). Cytonemes observed in the *Drosophila* wing disc correspond in length to the distribution of HH signaling and can also contain PTCH, raising the possibility that cytonemes can both send and receive signals (Bischoff et al., 2013; Gradilla et al., 2014). In the developing chick limb, cytonemes also contain HH, indicating that cytonemes may represent an evolutionarily conserved mechanism for distributing HH signals (Sanders et al., 2013). It will be of interest to specifically disrupt vertebrate cytonemes to assess how they shape HH signaling.

## The HH Receptor, PTCH, Transports Sterols

Beyond HH itself, constituents of the HH signal transduction pathway are intimately associated with lipids. The HH receptor is a twelve-pass transmembrane protein called Patched (PTCH), of which most vertebrates have two homologs, PTCH1 and PTCH2 (Ingham and McMahon, 2001). PTCH proteins form a clade of the larger resistance-nodulation-division (RND) transporter-like family (Taipale et al., 2002). Bacterial RND proteins are exporters of diverse molecules that include hopanoids, sterol-like molecules (Tseng et al., 1999). In addition to PTCH, the RND family includes NPC1, a transporter which in animals conducts cholesterol across the lysosomal membrane (Kwon et al., 2009). Like NPC1, PTCH includes a sterol-sensing domain (SSD), implicated in the subcellular trafficking of sterols. Another similarity to NPC1 is that PTCH1 contains a hydrophobic channel that may contain sterols (Gong et al., 2018).

These structural similarities suggest that PTCH1 functions similarly to NPC1, validated by several cryo-EM-elucidated structures of the core of PTCH1 (Qi et al., 2018a; Qi et al., 2018b; Gong et al., 2018; Qi et al., 2019; Qian et al., 2019; Rudolf et al., 2019). Indeed, PTCH1 can efflux a fluorescent form of cholesterol and SHH inhibition of PTCH1 increases intracellular cholesterol concentration (Bidet et al., 2011). Structural analysis reveals that PTCH1 interacts with sterols at ten or more sites and can partially lift sterols out of the membrane bilayer (Qi et al., 2019). Although the functional importance of the partial removal of a sterol from the membrane is unclear, it may represent an intermediate step in sterol transport. Indeed, PTCH1 can transport lipid sterols away from the inner leaflet of the membrane (Zhang et al., 2018; Qi et al., 2019) and it is likely that the binding of PTCH1 to HH blocks PTCH1 to allow buildup of a SMO-activating sterol, perhaps specifically in the ciliary membrane, thereby activating the downstream signal transduction pathway.

Numerous PTCH1 mutations associated with the human birth defect holoprosencephaly increase its ability to inhibit SMO (Petrov et al., 2021). Loss-of-function mutations in PTCH1 cause misactivation of SMO and some forms of cancer (Gailani et al., 1996; Hahn et al., 1996; Johnson et al., 1996). Whether either set of missense mutations alter sterol transport will be interesting to assess.

Other hints about PTCH function can be gleaned from evolutionary perspectives. Some bilateria, notably *Caenorhabditis elegans*, have lost the HH pathway but retained PTCH homologs. One of these, PTR-18 clears a secreted protein, GRL-7, distantly related to HH (Chiyoda et al., 2021), suggesting that PTCH can be repurposed to function independently of HH pathway regulation. Another *C. elegans* PTCH homolog, PTC-3, prevents intracellular cholesterol accumulation (Cadena del Castillo et al., 2021), further supporting the idea that PTCH family members are sterol transporters.

Interestingly, a paralog of PTCH cleverly called Dispatched1 (DISP1) functions not in HH reception but in transmitting HH from the cells in which it is produced (Burke et al., 1999). DISP1 forms a sodium channel and depends on the sodium gradient to release SHH from producing cells, raising the possibility that flux of sodium down its chemiosmotic gradient may power the extraction of cholesteroylated HH from the membrane (Petrov et al., 2020; Wang et al., 2021). Recent structures of DISP1 reveal that, like PTCH1, it partially displaces a sterol from the membrane bilayer (Wang et al., 2021). This lifted sterol may represent an ability of DISP1 to pry the cholesterol adduct of HH out of the plasma membrane, potentially a step in its transfer of HH to SCUBE2.

Many of the residues involved in coordinating sodium are also present in PTCH1, consistent with evidence that a sodium or potassium gradient is critical to the ability of PTCH1 to suppress the signaling activity of SMO (Myers et al., 2017; Petrov et al., 2020). It will be interesting to determine how PTCH1 uses a monovalent cation gradient. Perhaps cation flux through PTCH1 powers the removal of SMO-activating sterols from the ciliary membrane in a way that is analogous to RND-mediated export of hopanoids from the inner membrane of bacteria.

In addition to PTCH, HH is bound by additional proteins not essential for all HH communication, including HHIP, CDON, BOC, GAS1 and LDL receptor-related protein 2 (LRP2) (Chuang and McMahon, 1999; Stebel et al., 2000; Yao et al., 2006; Zhang et al., 2006; Christ et al., 2012). These auxiliary HH-binding proteins operate differently from each other: HHIP negatively regulates HH signaling while the others potentiate HH signaling (except for in the retina, where LRP2 inhibits HH signaling) (Christ et al., 2015).

As its name implies, LRP2 is a member of the family of low-density lipoprotein (LDL) receptors. LRP2 is required, like SHH, for forebrain development in mice (Willnow et al., 1996). Inherited mutations of *LRP2* in humans cause Donnai-Barrow syndrome, which includes craniofacial defects that may be related to altered HH signaling (Kantarci et al., 2007, 2008).

Some other LRP family members also function in developmental pathways. For example, LRP5 and LRP6 are part of the WNT receptor complex (Pinson et al., 2000). WNT ligands, like HH, are palmitoylated (Willert et al., 2003). The best studied member of the family, LDLR, binds and endocytoses LDL, bringing cholesterol into the cell. In addition to HH, LRP2 binds to a variety of ligands, including proteins that carry steroid-like molecules (Christensen et al., 1999; Nykjaer et al., 1999; Hammes et al., 2005).

Where do ciliary lipids come from? In animals, cholesterol is generated within the cytosol and endoplasmic reticulum (ER) or delivered via LDLs. Upon uptake, LDL is endocytosed and fused with lysosomes to release cholesterol for delivery to the plasma membrane (Brown and Goldstein, 1986). A key regulator of plasma membrane cholesterol content is NPC1, mutated in Neiman-Pick disease. Mice lacking NPC1 show decreased ciliogenesis and shortened cilia, with decreased HH signaling in the cerebellum, raising the possibility that NPC1 helps deliver cholesterol to the ciliary membrane (Canterini et al., 2017). However, NPC1 is not generally required for HH signaling, indicating that either there are NPC1-independent mechanisms of delivering cholesterol to the ciliary membrane or that NPC1-dependent ciliary cholesterol is not essential for HH pathway activation.

The endocytosis of a variety of lipid-associated proteins via LRP family members raises the possibility that internalization of extracellular lipids was the original role for these proteins. Although speculative, it is possible to imagine that extracytosolic lipid-binding proteins, functionally akin to the evolutionarily ancient tubular lipid-binding proteins (TULIPs) or the more recently evolved cholesterol carrier NPC2, might have facilitated lipid uptake (Wong and Levine, 2017). Perhaps upon acquisition of multicellularity and increased needs for cell-cell communication, these extracellular lipid-binding proteins became lipoprotein receptors and acquired new roles in information transmission. The genomes of the simple animals, such as *Trichoplax*, sea anemones and sponges, encode members of the LRP family member (e.g., TRIADDRAFT_27379, TRIADDRAFT_19424, A0A1X7TVZ2), suggesting that LRP proteins arose before porifera and placozoa split from each other early in the evolution of multicellular animals. Thus, it is possible that evolution acted on a system for lipid nutrient uptake, converting it into systems for cell-cell communication such as WNT and HH signaling.

Like LRP proteins, a canonical HH pathway is present in many basal animals, including sponges and sea anemones, but is absent from choanoflagellates and other single-celled eukaryotes (Figure 1). Despite the absence of the complete HH pathway in protists, PTCH homologs are present in some protist genomes, raising the intriguing possibility that PTCH is the most evolutionarily ancient member of the pathway and was subsequently co-opted for HH signal transduction. For example, *Chlamydomonas* possesses two PTCH orthologs (Cre02.g093500 and Cre12.g496350) which, unfortunately, have not been studied.

The main sterol in *Chlamydomonas* membranes is not cholesterol, but ergosterol (Gealt et al., 1981). It will be interesting to discover whether protist PTCH family members share the interaction with sterols with their metazoan cousins. As yeast NPC1 transports ergosterol and animal NPC1 transports cholesterol, it is possible that PTCH has similarly evolved to transport different sterols in different organisms. Interestingly, one *Chlamydomonas* flagellar lipid, an ergosterol endoperoxide, can inhibit mammalian HH signaling (Sever et al., 2016), raising the possibility that protist PTCH homologs could act on sterols with sufficient similarity to animal sterols that they can interact with the mammalian HH signal transduction pathway. Perhaps elucidating the functions of protist PTCH homologs will provide insights into the types of sterols transported by these elusive channels.

## Cholesterol and Oxysterols Can Activate Smoothened

PTCH suppresses the function of SMO, the central positive activator of the downstream HH signal transduction pathway. SMO is comprised of an N-terminal, extracellular cysteine-rich domain (CRD), an extracellular linker domain, a transmembrane heptahelical bundle (HHB), and a C-terminal cytosolic tail.

How might PTCH inhibit SMO activity? Previous hypotheses posited that PTCH directly binds to and sequesters SMO in a way that is relieved upon HH binding to PTCH (Stone et al., 1996; Murone et al., 1999). However, PTCH and SMO do not interact tightly and have distinct subcellular distributions, even in the primary cilium (Denef et al., 2000; Corbit et al., 2005; Rohatgi et al., 2007). Moreover, PTCH can inhibit SMO sub-stoichiometrically, with half-maximal pathway activity observed only when SMO was in 50-fold molar excess of PTCH (Taipale et al., 2002). These data, combined with the ability of PTCH to transport sterols (Zhang et al., 2018; Qi et al., 2019), suggests that PTCH may export a SMO-activating sterol.

**FIGURE 3:**
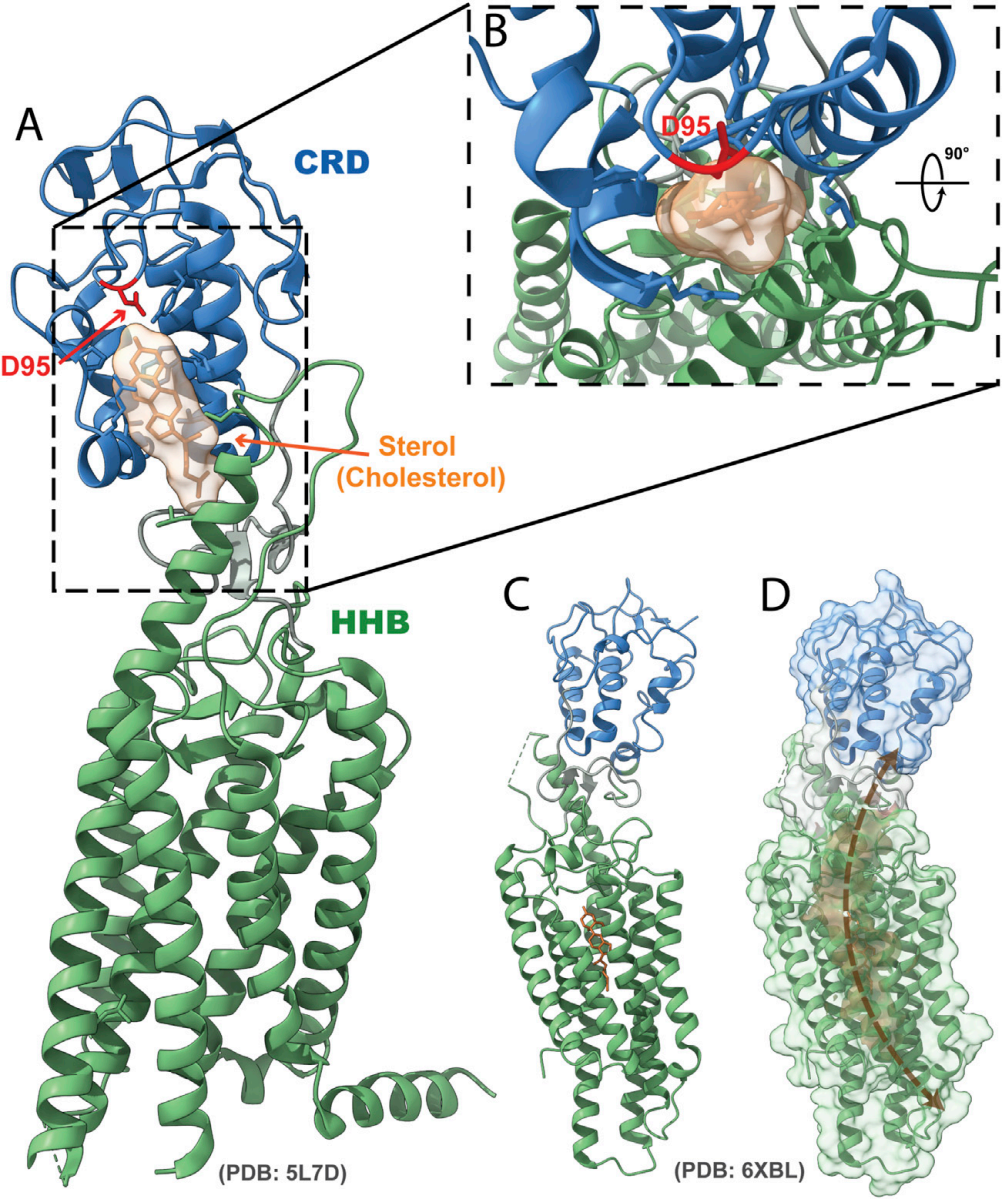
Structure of SMO bound to sterols. **(A)** Ribbon representation of the crystal structure of human SMO bound to a sterol in the CRD, with cholesterol depicted (PDB: 5L7D). Blue, CRD; green, HHB; orange, sterol; red, residue D95. **(B)** Inset depicts a top-down view of the CRD binding pocket with a sterol bound. **(C)** Structure of human SMO with one of the putative sterol-binding sites in the HHB depicted (PDB: 6XLB). **(D)** Surface rendering of the potential sterol channel in SMO.

Like PTCH, SMO binds sterols at several sites (Figures 3A,C) (Myers et al., 2013; Rana et al., 2013; Byrne et al., 2016; Huang et al., 2016, 2018; Luchetti et al., 2016; Raleigh et al., 2018; Deshpande et al., 2019). SMO mutations that alter individual sterol sites, either within the CRD or HHB, compromise HH signal transduction (Myers et al., 2013; Nachtergaele et al., 2013; Raleigh et al., 2018), suggesting that sterol binding is important for signal transduction.

How sterols activate SMO binding remains unclear. Although unprecedented for a GPCR-like protein, one possibility is that these binding sites form a continuous intramolecular channel through SMO capable of sterol transport (Figures 3C,D) (Huang et al., 2018; Qi et al., 2020). Mutations in the HHB that are likely to prevent sterol movement within SMO constitutively activate signaling (Qi et al., 2020). Perhaps these mutations block sterol transit through SMO and increased sterol occupancy within SMO is sufficient to activate SMO.

In addition to interacting with sterols, SMO can be covalently modified by cholesterol at the CRD (Xiao et al., 2017). This cholesterylation occurs in human SMO at the D95 residue (mouse SMO D99) within the CRD sterol binding site (Byrne et al., 2016) (Figures 3A,B). Mutation of this aspartic acid to hinder cholesterol modification of SMO compromises ciliary localization and signaling (Xiao et al., 2017; Hu et al., 2022). Thus, it is likely that covalent binding of SMO to cholesterol stabilizes its active state. It will be interesting to establish whether both non-covalent and covalent interaction with cholesterol are sufficient to promote pathway activity *in vivo*.

The cholesterylation of SMO is inhibited by PTCH1 and promoted by HH ligand (Xiao et al., 2017). Understanding where within the cell SMO is cholesterylated (e.g., before or after ciliary localization) will help reveal how sterols affect HH signaling. As SMO lacking the CRD domain is still able to weakly activate the downstream pathway (Raleigh et al., 2018), sterol interaction with the CRD is likely to be a modulatory effect on SMO activity.

Both oxysterols and cholesterol can bind SMO. However, it is still an open question which sterols activate SMO *in vivo*. Indeed, the SMO-activating sterol may be cholesterol, oxysterol, or some combination thereof. Given that cholesterol is a highly abundant lipid in the plasma membranes of animal cells and quickly transits between the inner and outer leaflets, it is unclear how PTCH1 could inhibit cholesterol accumulation specifically in the outer leaflet to prevent SMO misactivation. One possibility is that much of the membrane-associated cholesterol is sequestered as a form that cannot regulate SMO (Kinnebrew et al., 2019; Radhakrishnan et al., 2020). Thus, the pool of cholesterol relevant to SMO regulation (“accessible” cholesterol) may be smaller than the total cholesterol pool.

Certain oxysterols [e.g., 7β,27-DHC, 24k-C, and 24(S),25-EC] are enriched in the primary cilium, can bind to SMO, and can promote the accumulation of SMO in cilia, and thus are candidate regulators of SMO activation (Raleigh et al., 2018). As SMO possesses multiple sterol binding sites (Myers et al., 2013; Raleigh et al., 2018; Qi et al., 2020), multiple sterols may be relevant even to a single molecule of SMO. Indeed, it could even be possible that the same sterol could antagonize SMO function when binding at or near the orthosteric site within the heptahelical core and agonize SMO when binding the extracellular CRD.

## Conclusion

In this review, we have summarized the intimate connection between HH signaling and lipids. Lipids participate in the HH-mediated orchestration of developmental and homeostatic processes at multiple levels, including as constituents of cellular membranes, as ligands or substrates for key pathway components, and as covalent modifiers of HH and SMO.

Cilia are evolutionarily ancient organelles which possess a distinct ciliary lipid composition in organisms as diverse as *Chlamydomonas*, *Tetrahymena* and *Paramecia*. In vertebrates, primary cilia also have a unique lipid composition, including enrichment in PI(4)P. Vertebrate HH signal transduction depends on primary cilia, and on the lipids of the primary cilium, bringing a subcellular focus to many steps of HH signal transduction.

Despite remarkable advances, our understanding of the role and regulation of lipids trails our understanding of proteins. The development of new tools to detect and perturb specific lipids will diminish this gap. For example, specific and sensitive lipid biosensors will permit visualization of the spatial distribution of the ciliary lipid composition. To help unravel how lipids function in HH signaling, it will be particularly helpful to develop sterol biosensors, refine mass spectrometry-based lipidomic approaches, and create optogenetic or chemogenetic approaches to specifically deplete lipids in subcellular domains such as the primary cilium. Especially in the emerging era of superresolution microscopy, identification of lipid domains and how they are dynamically regulated may be in the offing. As we have some understanding of how HH signals remodel ciliary protein composition, it will be particularly interesting to assess whether HH signals also dynamically remodel the ciliary lipid composition to activate signaling.

## References

[B1] Amanai K. Jiang J. (2001). Distinct Roles of Central Missing and Dispatched in Sending the Hedgehog Signal. Development 128, 5119–5127. 10.1242/dev.128.24.5119 PubMed Abstract | 10.1242/dev.128.24.5119 | Google Scholar 11748147

[B2] Andrews D. Nelson D. L. (1979). Biochemical Studies of the Excitable Membrane of Paramecium Tetraurelia. II. Phospholipids of Ciliary and Other Membranes. Biochimica Biophysica Acta (BBA) - Biomembr. 550, 174–187. 10.1016/0005-2736(79)90205-0 PubMed Abstract | 10.1016/0005-2736(79)90205-0 | Google Scholar 758943

[B3] Babin P. J. Bogerd J. Kooiman F. P. van Marrewijk W. J. A. van der Horst D. J. (1999). Apolipophorin II/I, Apolipoprotein B, Vitellogenin, and Microsomal Triglyceride Transfer Protein Genes Are Derived from a Common Ancestor. J. Mol. Evol. 49, 150–160. 10.1007/PL00006528 PubMed Abstract | 10.1007/PL00006528 | Google Scholar 10368443

[B4] Bachmann V. A. Mayrhofer J. E. Ilouz R. Tschaikner P. Raffeiner P. Röck R. (2016). Gpr161 Anchoring of PKA Consolidates GPCR and cAMP Signaling. Proc. Natl. Acad. Sci. U.S.A. 113, 7786–7791. 10.1073/pnas.1608061113 PubMed Abstract | 10.1073/pnas.1608061113 | Google Scholar 27357676 PMC4948347

[B5] Balla T. Várnai P. (2002). Visualizing Cellular Phosphoinositide Pools with GFP-Fused Protein-Modules. Sci. STKE 2002. pl3–pl3. 10.1126/STKE.2002.125.PL3 PubMed Abstract | 10.1126/STKE.2002.125.PL3 | Google Scholar 11917154

[B6] Bangs F. Anderson K. V. (2017). Primary Cilia and Mammalian Hedgehog Signaling. Cold Spring Harb. Perspect. Biol. 9, a028175. 10.1101/cshperspect.a028175 PubMed Abstract | 10.1101/cshperspect.a028175 | Google Scholar 27881449 PMC5411695

[B7] Bidet M. Joubert O. Lacombe B. Ciantar M. Nehmé R. Mollat P. (2011). The Hedgehog Receptor Patched Is Involved in Cholesterol Transport. PLoS ONE 6, e23834. 10.1371/journal.pone.0023834 PubMed Abstract | 10.1371/journal.pone.0023834 | Google Scholar 21931618 PMC3169562

[B8] Bielas S. L. Silhavy J. L. Brancati F. Kisseleva M. V. Al-Gazali L. Sztriha L. (2009). Mutations in INPP5E, Encoding Inositol Polyphosphate-5-Phosphatase E, Link Phosphatidyl Inositol Signaling to the Ciliopathies. Nat. Genet. 419 41, 1032–1036. 10.1038/ng.423 PubMed Abstract | 10.1038/ng.423 | Google Scholar PMC2746682 19668216

[B9] Bischoff M. Gradilla A.-C. Seijo I. Andrés G. Rodríguez-Navas C. González-Méndez L. (2013). Cytonemes Are Required for the Establishment of a Normal Hedgehog Morphogen Gradient in Drosophila Epithelia. Nat. Cell Biol. 15, 1269–1281. 10.1038/ncb2856 PubMed Abstract | 10.1038/ncb2856 | Google Scholar 24121526 PMC3840581

[B10] Breslow D. K. Koslover E. F. Seydel F. Spakowitz A. J. Nachury M. V. (2013). An *In Vitro* Assay for Entry into Cilia Reveals Unique Properties of the Soluble Diffusion Barrier. J. Cell Biol. 203, 129–147. 10.1083/jcb.201212024 PubMed Abstract | 10.1083/jcb.201212024 | Google Scholar 24100294 PMC3798247

[B11] Brown M. S. Goldstein J. L. (19861979). A Receptor-Mediated Pathway for Cholesterol Homeostasis. Science 232, 34–47. 10.1126/science.3513311 PubMed Abstract | 10.1126/science.3513311 | Google Scholar 3513311

[B12] Buglino J. A. Resh M. D. (2008). Hhat Is a Palmitoylacyltransferase with Specificity for N-Palmitoylation of Sonic Hedgehog. J. Biol. Chem. 283, 22076–22088. 10.1074/jbc.M803901200 PubMed Abstract | 10.1074/jbc.M803901200 | Google Scholar 18534984 PMC2494920

[B13] Burke R. Nellen D. Bellotto M. Hafen E. Senti K.-A. Dickson B. J. (1999). Dispatched, a Novel Sterol-Sensing Domain Protein Dedicated to the Release of Cholesterol-Modified Hedgehog from Signaling Cells. Cell 99, 803–815. 10.1016/s0092-8674(00)81677-3 PubMed Abstract | 10.1016/s0092-8674(00)81677-3 | Google Scholar 10619433

[B14] Byrne E. F. X. Sircar R. Miller P. S. Hedger G. Luchetti G. Nachtergaele S. (2016). Structural Basis of Smoothened Regulation by its Extracellular Domains. Nature 535, 517–522. 10.1038/nature18934 PubMed Abstract | 10.1038/nature18934 | Google Scholar 27437577 PMC4970916

[B15] Cadena del Castillo C. E. Hannich J. T. Kaech A. Chiyoda H. Brewer J. Fukuyama M. (2021). Patched Regulates Lipid Homeostasis by Controlling Cellular Cholesterol Levels. Nat. Commun. 12, 4898. 10.1038/s41467-021-24995-9 PubMed Abstract | 10.1038/s41467-021-24995-9 | Google Scholar 34385431 PMC8361143

[B16] Callejo A. Torroja C. Quijada L. Guerrero I. (2006). Hedgehog Lipid Modifications Are Required for Hedgehog Stabilization in the Extracellular Matrix. Development 133, 471–483. 10.1242/dev.02217 PubMed Abstract | 10.1242/dev.02217 | Google Scholar 16396909

[B17] Canterini S. Dragotto J. Dardis A. Zampieri S. de Stefano M. E. Mangia F. (2017). Shortened Primary Cilium Length and Dysregulated Sonic Hedgehog Signaling in Niemann-Pick C1 Disease. Hum. Mol. Genet. 26, 2277–2289. 10.1093/hmg/ddx118 PubMed Abstract | 10.1093/hmg/ddx118 | Google Scholar 28379564

[B18] Chailley B. Boisvieux-Ulrich E. (1985). Detection of Plasma Membrane Cholesterol by Filipin during Microvillogenesis and Ciliogenesis in Quail Oviduct. J. Histochem Cytochem. 33, 1–10. 10.1177/33.1.3965567 PubMed Abstract | 10.1177/33.1.3965567 | Google Scholar 3965567

[B19] Chamoun Z. Mann R. K. Nellen D. von Kessler D. P. Bellotto M. Beachy P. A. (20011979). Skinny Hedgehog, an Acyltransferase Required for Palmitoylation and Activity of the Hedgehog Signal. Science 293, 2080–2084. 10.1126/science.1064437 PubMed Abstract | 10.1126/science.1064437 | Google Scholar 11486055

[B20] Chávez M. Ena S. Van Sande J. de Kerchove d’Exaerde A. Schurmans S. Schiffmann S. N. (2015). Modulation of Ciliary Phosphoinositide Content Regulates Trafficking and Sonic Hedgehog Signaling Output. Dev. Cell 34, 338–350. 10.1016/J.DEVCEL.2015.06.016 PubMed Abstract | 10.1016/J.DEVCEL.2015.06.016 | Google Scholar 26190144

[B21] Chen M.-H. Li Y.-J. Kawakami T. Xu S.-M. Chuang P.-T. (2004). Palmitoylation Is Required for the Production of a Soluble Multimeric Hedgehog Protein Complex and Long-Range Signaling in Vertebrates. Genes Dev. 18, 641–659. 10.1101/gad.1185804 PubMed Abstract | 10.1101/gad.1185804 | Google Scholar 15075292 PMC387240

[B22] Chiyoda H. Kume M. del Castillo C. C. Kontani K. Spang A. Katada T. (2021). *Caenorhabditis elegans* PTR/PTCHD PTR-18 Promotes the Clearance of Extracellular Hedgehog-Related Protein via Endocytosis. PLoS Genet. 17, e1009457. 10.1371/journal.pgen.1009457 PubMed Abstract | 10.1371/journal.pgen.1009457 | Google Scholar 33872306 PMC8104386

[B23] Christ A. Christa A. Klippert J. Eule J. C. Bachmann S. Wallace V. A. (2015). LRP2 Acts as SHH Clearance Receptor to Protect the Retinal Margin from Mitogenic Stimuli. Dev. Cell 35, 36–48. 10.1016/j.devcel.2015.09.001 PubMed Abstract | 10.1016/j.devcel.2015.09.001 | Google Scholar 26439398

[B24] Christ A. Christa A. Kur E. Lioubinski O. Bachmann S. Willnow T. E. (2012). LRP2 Is an Auxiliary SHH Receptor Required to Condition the Forebrain Ventral Midline for Inductive Signals. Dev. Cell 22, 268–278. 10.1016/j.devcel.2011.11.023 PubMed Abstract | 10.1016/j.devcel.2011.11.023 | Google Scholar 22340494

[B25] Christensen E. I. Moskaug J. Ø. Vorum H. Jacobsen C. Gundersen T. E. Nykjær A. (1999). Evidence for an Essential Role of Megalin in Transepithelial Transport of Retinol. J. Am. Soc. Nephrol. 10, 685–695. 10.1681/ASN.V104685 PubMed Abstract | 10.1681/ASN.V104685 | Google Scholar 10203351

[B26] Chuang P.-T. McMahon A. P. (1999). Vertebrate Hedgehog Signalling Modulated by Induction of a Hedgehog-Binding Protein. Nature 397, 617–621. 10.1038/17611 PubMed Abstract | 10.1038/17611 | Google Scholar 10050855

[B27] Conduit S. E. Davies E. M. Fulcher A. J. Oorschot V. Mitchell C. A. (2021). Superresolution Microscopy Reveals Distinct Phosphoinositide Subdomains within the Cilia Transition Zone. Front. Cell Dev. Biol. 9. 10.3389/fcell.2021.634649 PubMed Abstract | 10.3389/fcell.2021.634649 | Google Scholar PMC8120242 33996795

[B28] Conduit S. E. Vanhaesebroeck B. (2020). Phosphoinositide Lipids in Primary Cilia Biology. Biochem. J. 477, 3541–3565. 10.1042/BCJ20200277 PubMed Abstract | 10.1042/BCJ20200277 | Google Scholar 32970140 PMC7518857

[B29] Connor W. E. Lin D. S. Wolf D. P. Alexander M. (1998). Uneven Distribution of Desmosterol and Docosahexaenoic Acid in the Heads and Tails of Monkey Sperm. J. Lipid Res. 39, 1404–1411. 10.1016/s0022-2275(20)32521-9 PubMed Abstract | 10.1016/s0022-2275(20)32521-9 | Google Scholar 9684743

[B30] Cooper M. K. Wassif C. A. Krakowiak P. A. Taipale J. Gong R. Kelley R. I. (2003). A Defective Response to Hedgehog Signaling in Disorders of Cholesterol Biosynthesis. Nat. Genet. 33 (4), 508–513. 10.1038/ng1134 PubMed Abstract | 10.1038/ng1134 | Google Scholar 12652302

[B31] Corbit K. C. Aanstad P. Singla V. Norman A. R. Stainier D. Y. R. Reiter J. F. (2005). Vertebrate Smoothened Functions at the Primary Cilium. Nature 437, 1018–1021. 10.1038/nature04117 PubMed Abstract | 10.1038/nature04117 | Google Scholar 16136078

[B32] Coulter M. E. Dorobantu C. M. Lodewijk G. A. Delalande F. Cianferani S. Ganesh V. S. (2018). The ESCRT-III Protein CHMP1A Mediates Secretion of Sonic Hedgehog on a Distinctive Subtype of Extracellular Vesicles. Cell Rep. 24, 973–986. e8. 10.1016/j.celrep.2018.06.100 PubMed Abstract | 10.1016/j.celrep.2018.06.100 | Google Scholar 30044992 PMC6178983

[B33] Creanga A. Glenn T. D. Mann R. K. Saunders A. M. Talbot W. S. Beachy P. A. (2012). Scube/You Activity Mediates Release of Dually Lipid-Modified Hedgehog Signal in Soluble Form. Genes Dev. 26, 1312–1325. 10.1101/gad.191866.112 PubMed Abstract | 10.1101/gad.191866.112 | Google Scholar 22677548 PMC3387659

[B34] Das A. Brown M. S. Anderson D. D. Goldstein J. L. Radhakrishnan A. (2014). Three Pools of Plasma Membrane Cholesterol and Their Relation to Cholesterol Homeostasis. Elife 3. 10.7554/eLife.02882 10.7554/eLife.02882 | Google Scholar PMC4086274 24920391

[B35] Dawber R. J. Hebbes S. Herpers B. Docquier F. van den Heuvel M. (2005). Differential Range and Activity of Various Forms of the Hedgehog Protein. BMC Dev. Biol. 5, 21. 10.1186/1471-213X-5-21 PubMed Abstract | 10.1186/1471-213X-5-21 | Google Scholar 16197551 PMC1266354

[B36] Delling M. DeCaen P. G. Doerner J. F. Febvay S. Clapham D. E. (2013). Primary Cilia Are Specialized Calcium Signalling Organelles. Nature 504, 311–314. 10.1038/nature12833 PubMed Abstract | 10.1038/nature12833 | Google Scholar 24336288 PMC4112737

[B37] Denef N. Neubüser D. Perez L. Cohen S. M. (2000). Hedgehog Induces Opposite Changes in Turnover and Subcellular Localization of Patched and Smoothened. Cell 102, 521–531. 10.1016/S0092-8674(00)00056-8 PubMed Abstract | 10.1016/S0092-8674(00)00056-8 | Google Scholar 10966113

[B38] Deshpande I. Liang J. Hedeen D. Roberts K. J. Zhang Y. Ha B. (2019). Smoothened Stimulation by Membrane Sterols Drives Hedgehog Pathway Activity. Nature 571, 284–288. 10.1038/s41586-019-1355-4 PubMed Abstract | 10.1038/s41586-019-1355-4 | Google Scholar 31263273 PMC6709672

[B39] di Paolo G. de Camilli P. (2006). Phosphoinositides in Cell Regulation and Membrane Dynamics. Nature 443, 651–657. 10.1038/nature05185 PubMed Abstract | 10.1038/nature05185 | Google Scholar 17035995

[B40] Dwyer J. R. Sever N. Carlson M. Nelson S. F. Beachy P. A. Parhami F. (2007). Oxysterols Are Novel Activators of the Hedgehog Signaling Pathway in Pluripotent Mesenchymal Cells. J. Biol. Chem. 282, 8959–8968. 10.1074/JBC.M611741200 PubMed Abstract | 10.1074/JBC.M611741200 | Google Scholar 17200122

[B41] Dyson J. M. Conduit S. E. Feeney S. J. Hakim S. DiTommaso T. Fulcher A. J. (2017). INPP5E Regulates Phosphoinositide-dependent Cilia Transition Zone Function. J. Cell Biol. 216, 247–263. 10.1083/jcb.201511055 PubMed Abstract | 10.1083/jcb.201511055 | Google Scholar 27998989 PMC5223597

[B42] Eaton S. (2008). Multiple Roles for Lipids in the Hedgehog Signalling Pathway. Nat. Rev. Mol. Cell Biol. 9, 437–445. 10.1038/nrm2414 PubMed Abstract | 10.1038/nrm2414 | Google Scholar 18500255

[B43] Findakly S. Daggubati V. Garcia G. LaStella S. A. Choudhury A. Tran C. (2021). Sterol and Oxysterol Synthases Near the Ciliary Base Activate the Hedgehog Pathway. J. Cell Biol. 220. 10.1083/jcb.202002026 PubMed Abstract | 10.1083/jcb.202002026 | Google Scholar PMC7721912 33284321

[B44] Fitzky B. U. Witsch-Baumgartner M. Erdel M. Lee J. N. Paik Y.-K. Glossmann H. (1998). Mutations in the Δ7-sterol Reductase Gene in Patients with the Smith-Lemli-Opitz Syndrome. Proc. Natl. Acad. Sci. U.S.A. 95, 8181–8186. 10.1073/pnas.95.14.8181 PubMed Abstract | 10.1073/pnas.95.14.8181 | Google Scholar 9653161 PMC20950

[B45] Forbes A. J. Nakano Y. Taylor A. M. Ingham P. W. (1993). Genetic Analysis of *Hedgehog* Signalling in the *Drosophila* Embryo. Development 119, 115–124. 10.1242/dev.119.Supplement.115 10.1242/dev.119.Supplement.115 | Google Scholar 8049467

[B46] Forte M. Satow Y. Nelson D. Kung C. (1981). Mutational Alteration of Membrane Phospholipid Composition and Voltage-Sensitive Ion Channel Function in Paramecium. Proc. Natl. Acad. Sci. U.S.A. 78, 7195–7199. 10.1073/pnas.78.11.7195 PubMed Abstract | 10.1073/pnas.78.11.7195 | Google Scholar 6273919 PMC349223

[B47] Gailani M. R. Ståhle-Bäckdahl M. Leffell D. J. Glyn M. Zaphiropoulos P. G. Undén A. B. (1996). The Role of the Human Homologue of Drosophila Patched in Sporadic Basal Cell Carcinomas. Nat. Genet. 14, 78–81. 10.1038/ng0996-78 PubMed Abstract | 10.1038/ng0996-78 | Google Scholar 8782823

[B48] Gallet A. Rodriguez R. Ruel L. Therond P. P. (2003). Cholesterol Modification of Hedgehog Is Required for Trafficking and Movement, Revealing an Asymmetric Cellular Response to Hedgehog. Dev. Cell 4, 191–204. 10.1016/S1534-5807(03)00031-5 PubMed Abstract | 10.1016/S1534-5807(03)00031-5 | Google Scholar 12586063

[B49] Gallet A. Ruel L. Staccini-Lavenant L. Thérond P. P. (2006). Cholesterol Modification Is Necessary for Controlled Planar Long-Range Activity of Hedgehog in Drosophila Epithelia. Development 133, 407–418. 10.1242/dev.02212 PubMed Abstract | 10.1242/dev.02212 | Google Scholar 16396912

[B50] Garcia G. Raleigh D. R. Reiter J. F. (2018). How the Ciliary Membrane Is Organized Inside-Out to Communicate Outside-In. Curr. Biol. 28, R421–R434. 10.1016/j.cub.2018.03.010 PubMed Abstract | 10.1016/j.cub.2018.03.010 | Google Scholar 29689227 PMC6434934

[B51] Garcia-Gonzalo F. R. Phua S. C. Roberson E. C. Garcia G. Abedin M. Schurmans S. (2015). Phosphoinositides Regulate Ciliary Protein Trafficking to Modulate Hedgehog Signaling. Dev. Cell 34, 400–409. 10.1016/j.devcel.2015.08.001 PubMed Abstract | 10.1016/j.devcel.2015.08.001 | Google Scholar 26305592 PMC4557815

[B52] Garcia-Gonzalo F. R. Reiter J. F. (2017). Open Sesame: How Transition Fibers and the Transition Zone Control Ciliary Composition. Cold Spring Harb. Perspect. Biol. 9, a028134. 10.1101/cshperspect.a028134 PubMed Abstract | 10.1101/cshperspect.a028134 | Google Scholar 27770015 PMC5287074

[B53] Gealt M. A. Adler J. H. Nes W. R. (1981). The Sterols and Fatty Acids from Purified Flagella of Chlamydomonas Reinhardi. Lipids 16, 133–136. 10.1007/BF02535687 10.1007/BF02535687 | Google Scholar

[B54] Goetz J. A. Singh S. Suber L. M. Kull F. J. Robbins D. J. (2006). A Highly Conserved Amino-Terminal Region of Sonic Hedgehog Is Required for the Formation of its Freely Diffusible Multimeric Form. J. Biol. Chem. 281, 4087–4093. 10.1074/jbc.M511427200 PubMed Abstract | 10.1074/jbc.M511427200 | Google Scholar 16339763

[B55] Gong X. Qian H. Cao P. Zhao X. Zhou Q. Lei J. (2018). Structural Basis for the Recognition of Sonic Hedgehog by Human Patched1. Science 361, 361. 10.1126/science.aas8935 PubMed Abstract | 10.1126/science.aas8935 | Google Scholar 29954986

[B56] Gradilla A.-C. González E. Seijo I. Andrés G. Bischoff M. González-Mendez L. (2014). Exosomes as Hedgehog Carriers in Cytoneme-Mediated Transport and Secretion. Nat. Commun. 5. 10.1038/ncomms6649 PubMed Abstract | 10.1038/ncomms6649 | Google Scholar 25472772

[B57] Hahn H. Wicking C. Zaphiropoulos P. G. Gailani M. R. Shanley S. Chidambaram A. (1996). Mutations of the Human Homolog of Drosophila Patched in the Nevoid Basal Cell Carcinoma Syndrome. Cell 85, 841–851. 10.1016/S0092-8674(00)81268-4 PubMed Abstract | 10.1016/S0092-8674(00)81268-4 | Google Scholar 8681379

[B58] Hammes A. Andreassen T. K. Spoelgen R. Raila J. Hubner N. Schulz H. (2005). Role of Endocytosis in Cellular Uptake of Sex Steroids. Cell 122, 751–762. 10.1016/j.cell.2005.06.032 PubMed Abstract | 10.1016/j.cell.2005.06.032 | Google Scholar 16143106

[B59] Harayama T. Riezman H. (2018). Understanding the Diversity of Membrane Lipid Composition. Nat. Rev. Mol. Cell Biol. 19, 281–296. 10.1038/nrm.2017.138 PubMed Abstract | 10.1038/nrm.2017.138 | Google Scholar 29410529

[B60] Haycraft C. J. Banizs B. Aydin-Son Y. Zhang Q. Michaud E. J. Yoder B. K. (2005). Gli2 and Gli3 Localize to Cilia and Require the Intraflagellar Transport Protein Polaris for Processing and Function. PLoS Genet. preprint, e53. 10.1371/journal.pgen.001005310.1371/journal.pgen.0010053.eor PubMed Abstract | 10.1371/journal.pgen.001005310.1371/journal.pgen.0010053.eor | Google Scholar PMC1270009 16254602

[B61] Hilgendorf K. I. Johnson C. T. Mezger A. Rice S. L. Norris A. M. Demeter J. (2019). Omega-3 Fatty Acids Activate Ciliary FFAR4 to Control Adipogenesis. Cell 179, 1289–1305. e21. 10.1016/j.cell.2019.11.005 PubMed Abstract | 10.1016/j.cell.2019.11.005 | Google Scholar 31761534 PMC7332222

[B62] Hollway G. E. Maule J. Gautier P. Evans T. M. Keenan D. G. Lohs C. (2006). Scube2 Mediates Hedgehog Signalling in the Zebrafish Embryo. Dev. Biol. 294, 104–118. 10.1016/j.ydbio.2006.02.032 PubMed Abstract | 10.1016/j.ydbio.2006.02.032 | Google Scholar 16626681

[B63] Hu A. Zhang J.-Z. Wang J. Li C.-C. Yuan M. Deng G. (2022). Cholesterylation of Smoothened Is a Calcium-Accelerated Autoreaction Involving an Intramolecular Ester Intermediate. Cell Res. 32, 288–301. 10.1038/s41422-022-00622-0 PubMed Abstract | 10.1038/s41422-022-00622-0 | Google Scholar 35121857 PMC8888579

[B64] Huang P. Nedelcu D. Watanabe M. Jao C. Kim Y. Liu J. (2016). Cellular Cholesterol Directly Activates Smoothened in Hedgehog Signaling. Cell 166, 1176–1187. e14. 10.1016/j.cell.2016.08.003 PubMed Abstract | 10.1016/j.cell.2016.08.003 | Google Scholar 27545348 PMC5035717

[B65] Huang P. Zheng S. Wierbowski B. M. Kim Y. Nedelcu D. Aravena L. (2018). Structural Basis of Smoothened Activation in Hedgehog Signaling. Cell 174, 312–324. e16. 10.1016/j.cell.2018.04.029 PubMed Abstract | 10.1016/j.cell.2018.04.029 | Google Scholar 29804838 PMC6046275

[B66] Huangfu D. Anderson K. V. (2005). Cilia and Hedgehog Responsiveness in the Mouse. Proc. Natl. Acad. Sci. U.S.A. 102, 11325–11330. 10.1073/pnas.0505328102 PubMed Abstract | 10.1073/pnas.0505328102 | Google Scholar 16061793 PMC1183606

[B67] Huangfu D. Liu A. Rakeman A. S. Murcia N. S. Niswander L. Anderson K. V. (2003). Hedgehog Signalling in the Mouse Requires Intraflagellar Transport Proteins. Nature 426, 83–87. 10.1038/nature02061 PubMed Abstract | 10.1038/nature02061 | Google Scholar 14603322

[B68] Ingham P. W. (2018). From *Drosophila* Segmentation to Human Cancer Therapy. Development 145. 10.1242/dev.168898 PubMed Abstract | 10.1242/dev.168898 | Google Scholar 30413531

[B69] Ingham P. W. McMahon A. P. (2001). Hedgehog Signaling in Animal Development: Paradigms and Principles. Genes Dev. 15, 3059–3087. 10.1101/gad.938601 PubMed Abstract | 10.1101/gad.938601 | Google Scholar 11731473

[B70] Ishikawa H. Marshall W. F. (2011). Ciliogenesis: Building the Cell's Antenna. Nat. Rev. Mol. Cell Biol. 12, 222–234. 10.1038/nrm3085 PubMed Abstract | 10.1038/nrm3085 | Google Scholar 21427764

[B71] Jacoby M. Cox J. J. Gayral S. Hampshire D. J. Ayub M. Blockmans M. (20092009). INPP5E Mutations Cause Primary Cilium Signaling Defects, Ciliary Instability and Ciliopathies in Human and Mouse. Nat. Genet. 41 (9), 1027–1031. 10.1038/ng.427 PubMed Abstract | 10.1038/ng.427 | Google Scholar 19668215

[B72] Jia J. Tong C. Wang B. Luo L. Jiang J. (20042005). Hedgehog Signalling Activity of Smoothened Requires Phosphorylation by Protein Kinase A and Casein Kinase I. Nature 432, 1045–1050. 10.1038/nature03179 PubMed Abstract | 10.1038/nature03179 | Google Scholar 15616566

[B73] Johnson J.-L. F. A. Hall T. E. Dyson J. M. Sonntag C. Ayers K. Berger S. (2012). Scube Activity Is Necessary for Hedgehog Signal Transduction *In Vivo* . Dev. Biol. 368, 193–202. 10.1016/j.ydbio.2012.05.007 PubMed Abstract | 10.1016/j.ydbio.2012.05.007 | Google Scholar 22609552

[B74] Johnson R. L. Rothman A. L. Xie J. Goodrich L. V. Bare J. W. Bonifas J. M. (1996). Human Homolog of Patched, a Candidate Gene for the Basal Cell Nevus Syndrome. Science 272, 1668–1671. 10.1126/science.272.5268.1668 PubMed Abstract | 10.1126/science.272.5268.1668 | Google Scholar 8658145

[B75] Kaneshiro E. S. Matesic D. F. Jayasimhulu K. (1984). Characterizations of Six Ethanolamine Sphingophospholipids from Paramecium Cells and Cilia. J. Lipid Res. 25, 369–377. 10.1016/S0022-2275(20)37810-X PubMed Abstract | 10.1016/S0022-2275(20)37810-X | Google Scholar 6202812

[B76] Kantarci S. Al-Gazali L. Hill R. S. Donnai D. Black G. C. M. Bieth E. (2007). Mutations in LRP2, Which Encodes the Multiligand Receptor Megalin, Cause Donnai-Barrow and Facio-Oculo-Acoustico-Renal Syndromes. Nat. Genet. 39, 957–959. 10.1038/ng2063 PubMed Abstract | 10.1038/ng2063 | Google Scholar 17632512 PMC2891728

[B77] Kantarci S. Ragge N. K. Thomas N. S. Robinson D. O. Noonan K. M. Russell M. K. (2008). Donnai-Barrow Syndrome (DBS/FOAR) in a Child with a homozygousLRP2mutation Due to Complete Chromosome 2 Paternal Isodisomy. Am. J. Med. Genet. 146A, 1842–1847. 10.1002/ajmg.a.32381 PubMed Abstract | 10.1002/ajmg.a.32381 | Google Scholar 18553518 PMC2891749

[B78] Kawakami A. Nojima Y. Toyoda A. Takahoko M. Satoh M. Tanaka H. (2005). The Zebrafish-Secreted Matrix Protein You/Scube2 Is Implicated in Long-Range Regulation of Hedgehog Signaling. Curr. Biol. 15, 480–488. 10.1016/j.cub.2005.02.018 PubMed Abstract | 10.1016/j.cub.2005.02.018 | Google Scholar 15753045

[B79] Kennedy K. E. Thompson G. A. (1970). Phosphonolipids: Localization in Surface Membranes of *Tetrahymena* . Science 168, 989–991. 10.1126/science.168.3934.989 PubMed Abstract | 10.1126/science.168.3934.989 | Google Scholar 5441031

[B80] Kinnebrew M. Iverson E. J. Patel B. B. Pusapati G. V. Kong J. H. Johnson K. A. (2019). Cholesterol Accessibility at the Ciliary Membrane Controls Hedgehog Signaling. Elife 8. 10.7554/eLife.50051 PubMed Abstract | 10.7554/eLife.50051 | Google Scholar PMC6850779 31657721

[B180] Kinnebrew M. Luchetti G. Sircar R. Frigui S. Viti L. V. Naito T. (2021). Patched 1 Reduces the Accessibility of Cholesterol in the Outer Leaflet of Membranes. Elife 10. 10.7554/eLife.70504 10.7554/eLife.70504 | Google Scholar PMC8654371 34698632

[B81] Kinsky S. C. Luse S. A. van Deenen L. L. (1966). Interaction of Polyene Antibiotics with Natural and Artificial Membrane Systems. Fed. Proc. 25, 1503–1510. Available at: http://www.ncbi.nlm.nih.gov/pubmed/5332190. PubMed Abstract | Google Scholar 5332190

[B82] Kong J. H. Siebold C. Rohatgi R. (2019). Biochemical Mechanisms of Vertebrate Hedgehog Signaling. Development 146 (10), dev166892. 10.1242/dev.166892 PubMed Abstract | 10.1242/dev.166892 | Google Scholar 31092502 PMC6550017

[B83] Kornberg T. B. (2014). The Contrasting Roles of Primary Cilia and Cytonemes in Hh Signaling. Dev. Biol. 394, 1–5. 10.1016/j.ydbio.2014.07.015 PubMed Abstract | 10.1016/j.ydbio.2014.07.015 | Google Scholar 25072627 PMC4163518

[B84] Kuzhandaivel A. Schultz S. W. Alkhori L. Alenius M. (2014). Cilia-Mediated Hedgehog Signaling in Drosophila. Cell Rep. 7, 672–680. 10.1016/j.celrep.2014.03.052 PubMed Abstract | 10.1016/j.celrep.2014.03.052 | Google Scholar 24768000

[B85] Kwon H. J. Abi-Mosleh L. Wang M. L. Deisenhofer J. Goldstein J. L. Brown M. S. (2009). Structure of N-Terminal Domain of NPC1 Reveals Distinct Subdomains for Binding and Transfer of Cholesterol. Cell 137, 1213–1224. 10.1016/j.cell.2009.03.049 PubMed Abstract | 10.1016/j.cell.2009.03.049 | Google Scholar 19563754 PMC2739658

[B86] Leathes J. B. (1925). Croonian Lectures on the Rôle of Fats in Vital Phenomena. Lancet 205, 853–856. 10.1016/s0140-6736(01)22310-1 10.1016/s0140-6736(01)22310-1 | Google Scholar

[B87] Lee J. D. Kraus P. Gaiano N. Nery S. Kohtz J. Fishell G. (2001). An Acylatable Residue of Hedgehog Is Differentially Required in Drosophila and Mouse Limb Development. Dev. Biol. 233, 122–136. 10.1006/dbio.2001.0218 PubMed Abstract | 10.1006/dbio.2001.0218 | Google Scholar 11319862

[B88] Lee J. Ekker S. von Kessler D. Porter J. Sun B. Beachy P. (1994). Autoproteolysis in Hedgehog Protein Biogenesis. Science 266, 1528–1537. 10.1126/science.7985023 PubMed Abstract | 10.1126/science.7985023 | Google Scholar 7985023

[B89] Lewis P. M. Dunn M. P. McMahon J. A. Logan M. Martin J. F. St-Jacques B. (2001). Cholesterol Modification of Sonic Hedgehog Is Required for Long-Range Signaling Activity and Effective Modulation of Signaling by Ptc1. Cell 105, 599–612. 10.1016/S0092-8674(01)00369-5 PubMed Abstract | 10.1016/S0092-8674(01)00369-5 | Google Scholar 11389830

[B90] Li Y. Zhang H. Litingtung Y. Chiang C. (2006). Cholesterol Modification Restricts the Spread of Shh Gradient in the Limb Bud. Proc. Natl. Acad. Sci. U.S.A. 103, 6548–6553. 10.1073/pnas.0600124103 PubMed Abstract | 10.1073/pnas.0600124103 | Google Scholar 16611729 PMC1458921

[B91] Lobasso S. Lopalco P. Angelini R. Baronio M. Fanizzi F. P. Babudri F. (2010). Lipidomic Analysis of Porcine Olfactory Epithelial Membranes and Cilia. Lipids 45, 593–602. 10.1007/S11745-010-3432-1/FIGURES/6 PubMed Abstract | 10.1007/S11745-010-3432-1/FIGURES/6 | Google Scholar 20512424

[B92] Luchetti G. Sircar R. Kong J. H. Nachtergaele S. Sagner A. Byrne E. F. (2016). Cholesterol Activates the G-Protein Coupled Receptor Smoothened to Promote Hedgehog Signaling. Elife 5. 10.7554/ELIFE.20304 10.7554/ELIFE.20304 | Google Scholar PMC5123864 27705744

[B93] Luo N. Kumar A. Conwell M. Weinreb R. N. Anderson R. Sun Y. (2013). Compensatory Role of Inositol 5-Phosphatase INPP5B to OCRL in Primary Cilia Formation in Oculocerebrorenal Syndrome of Lowe. PLOS ONE 8, e66727. 10.1371/JOURNAL.PONE.0066727 PubMed Abstract | 10.1371/JOURNAL.PONE.0066727 | Google Scholar 23805271 PMC3689662

[B94] Luo N. West C. C. Murga-Zamalloa C. A. Sun L. Anderson R. M. Wells C. D. (2012). OCRL Localizes to the Primary Cilium: a New Role for Cilia in Lowe Syndrome. Hum. Mol. Genet. 21, 3333–3344. 10.1093/HMG/DDS163 PubMed Abstract | 10.1093/HMG/DDS163 | Google Scholar 22543976 PMC3392109

[B95] Maekawa M. Fairn G. D. (2014). Molecular Probes to Visualize the Location, Organization and Dynamics of Lipids. J. Cell Sci. 127, 4801–4812. 10.1242/jcs.150524 PubMed Abstract | 10.1242/jcs.150524 | Google Scholar 25179600

[B96] Marley A. Choy R. W.-Y. von Zastrow M. (2013). GPR88 Reveals a Discrete Function of Primary Cilia as Selective Insulators of GPCR Cross-Talk. PLoS ONE 8, e70857. 10.1371/journal.pone.0070857 PubMed Abstract | 10.1371/journal.pone.0070857 | Google Scholar 23936473 PMC3732291

[B97] Matsumoto Y. Morishima K.-I. Honda A. Watabe S. Yamamoto M. Hara M. (2005). R352Q Mutation of the DHCR7 Gene Is Common Among Japanese Smith-Lemli-Opitz Syndrome Patients. J. Hum. Genet. 50, 353–356. 10.1007/s10038-005-0267-3 PubMed Abstract | 10.1007/s10038-005-0267-3 | Google Scholar 16044199

[B98] Matusek T. Wendler F. Polès S. Pizette S. D’Angelo G. Fürthauer M. (2014). The ESCRT Machinery Regulates the Secretion and Long-Range Activity of Hedgehog. Nature 516, 99–103. 10.1038/nature13847 PubMed Abstract | 10.1038/nature13847 | Google Scholar 25471885

[B99] McConnell H. M. Radhakrishnan A. (2003). Condensed Complexes of Cholesterol and Phospholipids. Biochimica Biophysica Acta (BBA) - Biomembr. 1610, 159–173. 10.1016/S0005-2736(03)00015-4 10.1016/S0005-2736(03)00015-4 | Google Scholar 12648771

[B100] Melkonian M. Robenek H. Rassat J. (1982). Flagellar Membrane Specializations and Their Relationship to Mastigonemes and Microtubules in Euglena Gracilis. J. Cell Sci. 55, 115–135. 10.1242/jcs.55.1.115 PubMed Abstract | 10.1242/jcs.55.1.115 | Google Scholar 6809773

[B101] Micchelli C. A. The I. Selva E. Mogila V. Perrimon N. (2002). Rasp, a Putative Transmembrane Acyltransferase, Is Required for Hedgehog Signaling. Development 129, 843–851. 10.1242/dev.129.4.843 PubMed Abstract | 10.1242/dev.129.4.843 | Google Scholar 11861468

[B102] Mick D. U. Rodrigues R. B. Leib R. D. Adams C. M. Chien A. S. Gygi S. P. (2015). Proteomics of Primary Cilia by Proximity Labeling. Dev. Cell 35, 497–512. 10.1016/j.devcel.2015.10.015 PubMed Abstract | 10.1016/j.devcel.2015.10.015 | Google Scholar 26585297 PMC4662609

[B103] Miyamoto T. Hosoba K. Itabashi T. Iwane A. H. Akutsu S. N. Ochiai H. (2020). Insufficiency of Ciliary Cholesterol in Hereditary Zellweger Syndrome. Embo J. 39. 10.15252/embj.2019103499 PubMed Abstract | 10.15252/embj.2019103499 | Google Scholar PMC7298307 32368833

[B104] Mourvaki E. Cardinali R. Roberti R. Dal Bosco A. Castellini C. (2010). Desmosterol, the Main Sterol in Rabbit Semen: Distribution Among Semen Subfractions and its Role in the *In Vitro* Spermatozoa Acrosome Reaction and Motility. Asian J. Androl. 12, 862–870. 10.1038/aja.2010.25 PubMed Abstract | 10.1038/aja.2010.25 | Google Scholar 20729867 PMC3739068

[B105] Mukhopadhyay S. Badgandi H. B. Hwang S.-H. Somatilaka B. Shimada I. S. Pal K. (2017). Trafficking to the Primary Cilium Membrane. MBoC 28, 233–239. 10.1091/MBC.E16-07-0505/ASSET/IMAGES/LARGE/233FIG1 PubMed Abstract | 10.1091/MBC.E16-07-0505/ASSET/IMAGES/LARGE/233FIG1 | Google Scholar 28082521 PMC5231892

[B106] Mukhopadhyay S. Wen X. Chih B. Nelson C. D. Lane W. S. Scales S. J. (2010). TULP3 Bridges the IFT-A Complex and Membrane Phosphoinositides to Promote Trafficking of G Protein-Coupled Receptors into Primary Cilia. Genes Dev. 24, 2180–2193. 10.1101/GAD.1966210 PubMed Abstract | 10.1101/GAD.1966210 | Google Scholar 20889716 PMC2947770

[B107] Mukhopadhyay S. Wen X. Ratti N. Loktev A. Rangell L. Scales S. J. (2013). The Ciliary G-Protein-Coupled Receptor Gpr161 Negatively Regulates the Sonic Hedgehog Pathway via cAMP Signaling. Cell 152, 210–223. 10.1016/j.cell.2012.12.026 PubMed Abstract | 10.1016/j.cell.2012.12.026 | Google Scholar 23332756

[B108] Murone M. Rosenthal A. de Sauvage F. J. (1999). Sonic Hedgehog Signaling by the Patched-Smoothened Receptor Complex. Curr. Biol. 9, 76–84. 10.1016/S0960-9822(99)80018-9 PubMed Abstract | 10.1016/S0960-9822(99)80018-9 | Google Scholar 10021362

[B109] Myers B. R. Neahring L. Zhang Y. Roberts K. J. Beachy P. A. (2017). Rapid, Direct Activity Assays for Smoothened Reveal Hedgehog Pathway Regulation by Membrane Cholesterol and Extracellular Sodium. Proc. Natl. Acad. Sci. U.S.A. 114, E11141–E11150. 10.1073/PNAS.1717891115 PubMed Abstract | 10.1073/PNAS.1717891115 | Google Scholar 29229834 PMC5748227

[B110] Myers B. R. Sever N. Chong Y. C. Kim J. Belani J. D. Rychnovsky S. (2013). Hedgehog Pathway Modulation by Multiple Lipid Binding Sites on the Smoothened Effector of Signal Response. Dev. Cell 26, 346–357. 10.1016/j.devcel.2013.07.015 PubMed Abstract | 10.1016/j.devcel.2013.07.015 | Google Scholar 23954590 PMC4196939

[B111] Nachtergaele S. Whalen D. M. Mydock L. K. Zhao Z. Malinauskas T. Krishnan K. (2013). Structure and Function of the Smoothened Extracellular Domain in Vertebrate Hedgehog Signaling. Elife 2. 10.7554/eLife.01340 PubMed Abstract | 10.7554/eLife.01340 | Google Scholar PMC3809587 24171105

[B112] Nachury M. V. (2014). How Do Cilia Organize Signalling Cascades? Phil. Trans. R. Soc. B 369, 20130465. 10.1098/rstb.2013.0465 PubMed Abstract | 10.1098/rstb.2013.0465 | Google Scholar 25047619 PMC4113109

[B113] Nachury M. V. Mick D. U. (2019). Establishing and Regulating the Composition of Cilia for Signal Transduction. Nat. Rev. Mol. Cell Biol. 20, 389–405. 10.1038/s41580-019-0116-4 PubMed Abstract | 10.1038/s41580-019-0116-4 | Google Scholar 30948801 PMC6738346

[B114] Nechipurenko I. V. (2020). The Enigmatic Role of Lipids in Cilia Signaling. Front. Cell Dev. Biol. 8, 777. 10.3389/FCELL.2020.00777 PubMed Abstract | 10.3389/FCELL.2020.00777 | Google Scholar 32850869 PMC7431879

[B115] Nedelcu D. Liu J. Xu Y. Jao C. Salic A. (2013). Oxysterol Binding to the Extracellular Domain of Smoothened in Hedgehog Signaling. Nat. Chem. Biol. 9, 557–564. 10.1038/nchembio.1290 PubMed Abstract | 10.1038/nchembio.1290 | Google Scholar 23831757 PMC3749252

[B116] Nelson L. D. Johnson A. E. London E. (2008). How Interaction of Perfringolysin O with Membranes Is Controlled by Sterol Structure, Lipid Structure, and Physiological Low pH. J. Biol. Chem. 283, 4632–4642. 10.1074/jbc.M709483200 PubMed Abstract | 10.1074/jbc.M709483200 | Google Scholar 18089559

[B117] Nowaczyk M. J. M. Irons M. B. (2012). Smith-Lemli-Opitz Syndrome: Phenotype, Natural History, and Epidemiology. Am. J. Med. Genet. 160C, 250–262. 10.1002/ajmg.c.31343 PubMed Abstract | 10.1002/ajmg.c.31343 | Google Scholar 23059950

[B118] Nüsslein-Volhard C. Wieschaus E. Kluding H. (1984). Mutations Affecting the Pattern of the Larval Cuticle inDrosophila Melanogaster. Wilhelm Roux' Arch. 193, 267–282. 10.1007/BF00848156 10.1007/BF00848156 | Google Scholar 28305337

[B119] Nüsslein-Volhard C. Wieschaus E. (1980). Mutations Affecting Segment Number and Polarity in Drosophila. Nature 287, 795–801. 10.1038/287795a0 PubMed Abstract | 10.1038/287795a0 | Google Scholar 6776413

[B120] Nykjaer A. Dragun D. Walther D. Vorum H. Jacobsen C. Herz J. (1999). An Endocytic Pathway Essential for Renal Uptake and Activation of the Steroid 25-(OH) Vitamin D3. Cell 96, 507–515. 10.1016/S0092-8674(00)80655-8 PubMed Abstract | 10.1016/S0092-8674(00)80655-8 | Google Scholar 10052453

[B121] Palm W. Swierczynska M. M. Kumari V. Ehrhart-Bornstein M. Bornstein S. R. Eaton S. (2013). Secretion and Signaling Activities of Lipoprotein-Associated Hedgehog and Non-sterol-modified Hedgehog in Flies and Mammals. PLoS Biol. 11, e1001505. 10.1371/journal.pbio.1001505 PubMed Abstract | 10.1371/journal.pbio.1001505 | Google Scholar 23554573 PMC3595218

[B122] Panáková D. Sprong H. Marois E. Thiele C. Eaton S. (2005). Lipoprotein Particles Are Required for Hedgehog and Wingless Signalling. Nature 435, 58–65. 10.1038/nature03504 PubMed Abstract | 10.1038/nature03504 | Google Scholar 15875013

[B123] Pepinsky R. B. Zeng C. Wen D. Rayhorn P. Baker D. P. Williams K. P. (1998). Identification of a Palmitic Acid-Modified Form of Human Sonic Hedgehog. J. Biol. Chem. 273, 14037–14045. 10.1074/jbc.273.22.14037 PubMed Abstract | 10.1074/jbc.273.22.14037 | Google Scholar 9593755

[B124] Petrov K. de Almeida Magalhaes T. Salic A. (2021). Mechanism and Ultrasensitivity in Hedgehog Signaling Revealed by Patched1 Disease Mutations. Proc. Natl. Acad. Sci. U.S.A. 118. 10.1073/pnas.2006800118 10.1073/pnas.2006800118 | Google Scholar PMC8017988 33526656

[B125] Petrov K. Wierbowski B. M. Liu J. Salic A. (2020). Distinct Cation Gradients Power Cholesterol Transport at Different Key Points in the Hedgehog Signaling Pathway. Dev. Cell 55, 314–327. e7. 10.1016/j.devcel.2020.08.002 PubMed Abstract | 10.1016/j.devcel.2020.08.002 | Google Scholar 32860743 PMC7658045

[B126] Pinson K. I. Brennan J. Monkley S. Avery B. J. Skarnes W. C. (2000). An LDL-Receptor-Related Protein Mediates Wnt Signalling in Mice. Nature 407, 535–538. 10.1038/35035124 PubMed Abstract | 10.1038/35035124 | Google Scholar 11029008

[B127] Porter J. A. Ekker S. C. Park W.-J. von Kessler D. P. Young K. E. Chen C.-H. (1996a). Hedgehog Patterning Activity: Role of a Lipophilic Modification Mediated by the Carboxy-Terminal Autoprocessing Domain. Cell 86, 21–34. 10.1016/s0092-8674(00)80074-4 PubMed Abstract | 10.1016/s0092-8674(00)80074-4 | Google Scholar 8689684

[B128] Porter J. A. Young K. E. Beachy P. A. (1996b). Cholesterol Modification of Hedgehog Signaling Proteins in Animal Development. Science 274, 255–259. 10.1126/science.274.5285.255 PubMed Abstract | 10.1126/science.274.5285.255 | Google Scholar 8824192

[B129] Qi C. di Minin G. Vercellino I. Wutz A. Korkhov V. M. (2019). Structural Basis of Sterol Recognition by Human Hedgehog Receptor PTCH1. Sci. Adv. 5. 10.1126/sciadv.aaw6490 PubMed Abstract | 10.1126/sciadv.aaw6490 | Google Scholar PMC6750913 31555730

[B130] Qi X. Friedberg L. de Bose-Boyd R. Long T. Li X. (2020). Sterols in an Intramolecular Channel of Smoothened Mediate Hedgehog Signaling. Nat. Chem. Biol. 16, 1368–1375. 10.1038/s41589-020-0646-2 PubMed Abstract | 10.1038/s41589-020-0646-2 | Google Scholar 32929279 PMC7669734

[B131] Qi X. Schmiege P. Coutavas E. Li X. (2018a). Two Patched Molecules Engage Distinct Sites on Hedgehog Yielding a Signaling-Competent Complex. Science 362, 362. 10.1126/science.aas8843 PubMed Abstract | 10.1126/science.aas8843 | Google Scholar PMC6341491 30139912

[B132] Qi X. Schmiege P. Coutavas E. Wang J. Li X. (2018b). Structures of Human Patched and its Complex with Native Palmitoylated Sonic Hedgehog. Nature 560, 128–132. 10.1038/s41586-018-0308-7 PubMed Abstract | 10.1038/s41586-018-0308-7 | Google Scholar 29995851 PMC6341490

[B133] Qian H. Cao P. Hu M. Gao S. Yan N. Gong X. (2019). Inhibition of Tetrameric Patched1 by Sonic Hedgehog through an Asymmetric Paradigm. Nat. Commun. 10, 2320. 10.1038/s41467-019-10234-9 PubMed Abstract | 10.1038/s41467-019-10234-9 | Google Scholar 31127104 PMC6534611

[B134] Quirk J. van den Heuvel M. Henrique D. Marigo V. Jones T. A. Tabin C. (1997). The Smoothened Gene and Hedgehog Signal Transduction in Drosophila and Vertebrate Development. Cold Spring Harb. Symp. Quant. Biol. 62, 217–226. 10.1101/SQB.1997.062.01.027 PubMed Abstract | 10.1101/SQB.1997.062.01.027 | Google Scholar 9598354

[B135] Radhakrishnan A. Rohatgi R. Siebold C. (2020). Cholesterol Access in Cellular Membranes Controls Hedgehog Signaling. Nat. Chem. Biol. 16, 1303–1313. 10.1038/s41589-020-00678-2 PubMed Abstract | 10.1038/s41589-020-00678-2 | Google Scholar 33199907 PMC7872078

[B136] Raleigh D. R. Sever N. Choksi P. K. Sigg M. A. Hines K. M. Thompson B. M. (2018). Cilia-Associated Oxysterols Activate Smoothened. Mol. Cell 72, 316–327. e5. 10.1016/j.molcel.2018.08.034 PubMed Abstract | 10.1016/j.molcel.2018.08.034 | Google Scholar 30340023 PMC6503851

[B137] Rana R. Carroll C. E. Lee H.-J. Bao J. Marada S. Grace C. R. R. (2013). Structural Insights into the Role of the Smoothened Cysteine-Rich Domain in Hedgehog Signalling. Nat. Commun. 4, 2965. 10.1038/ncomms3965 PubMed Abstract | 10.1038/ncomms3965 | Google Scholar 24351982 PMC3890372

[B138] Reiter J. F. Leroux M. R. (2017). Genes and Molecular Pathways Underpinning Ciliopathies. Nat. Rev. Mol. Cell Biol. 18, 533–547. 10.1038/nrm.2017.60 PubMed Abstract | 10.1038/nrm.2017.60 | Google Scholar 28698599 PMC5851292

[B139] Rohatgi R. Milenkovic L. Scott M. P. (2007). Patched1 Regulates Hedgehog Signaling at the Primary Cilium. Science 317, 372–376. 10.1126/science.1139740 PubMed Abstract | 10.1126/science.1139740 | Google Scholar 17641202

[B140] Rudolf A. F. Kinnebrew M. Kowatsch C. Ansell T. B. el Omari K. Bishop B. (2019). The Morphogen Sonic Hedgehog Inhibits its Receptor Patched by a Pincer Grasp Mechanism. Nat. Chem. Biol. 15, 975–982. 10.1038/s41589-019-0370-y PubMed Abstract | 10.1038/s41589-019-0370-y | Google Scholar 31548691 PMC6764859

[B141] Sanders T. A. Llagostera E. Barna M. (2013). Specialized Filopodia Direct Long-Range Transport of SHH during Vertebrate Tissue Patterning. Nature 497, 628–632. 10.1038/nature12157 PubMed Abstract | 10.1038/nature12157 | Google Scholar 23624372 PMC4197975

[B142] Santos N. Reiter J. F. (2014). A Central Region of Gli2 Regulates its Localization to the Primary Cilium and Transcriptional Activity. J. Cell Sci. 127 (Pt 7), 1500–1510. 10.1242/jcs.139253 PubMed Abstract | 10.1242/jcs.139253 | Google Scholar 24463817 PMC3970560

[B143] Schink K. O. Tan K.-W. Stenmark H. (2016). Phosphoinositides in Control of Membrane Dynamics. Annu. Rev. Cell Dev. Biol. 32, 143–171. 10.1146/ANNUREV-CELLBIO-111315-125349 PubMed Abstract | 10.1146/ANNUREV-CELLBIO-111315-125349 | Google Scholar 27576122

[B144] Sever N. Mann R. K. Xu L. Snell W. J. Hernandez-Lara C. I. Porter N. A. (2016). Endogenous B-Ring Oxysterols Inhibit the Hedgehog Component Smoothened in a Manner Distinct from Cyclopamine or Side-Chain Oxysterols. Proc. Natl. Acad. Sci. U.S.A. 113, 5904–5909. 10.1073/pnas.1604984113 PubMed Abstract | 10.1073/pnas.1604984113 | Google Scholar PMC4889404 27162362

[B145] Shewan A. Eastburn D. J. Mostov K. (2011). Phosphoinositides in Cell Architecture. Cold Spring Harb. Perspect. Biol. 3, a004796. 10.1101/cshperspect.a004796 PubMed Abstract | 10.1101/cshperspect.a004796 | Google Scholar 21576256 PMC3140688

[B146] Smith J. D. Snyder W. R. Law J. H. (1970). Phosphonolipids in Tetrahymena Cilia. Biochem. Biophysical Res. Commun. 39, 1163–1169. 10.1016/0006-291X(70)90682-0 PubMed Abstract | 10.1016/0006-291X(70)90682-0 | Google Scholar 5513252

[B147] Souto-Padrón T. de Souza W. (1986). The Surface Charge of Trypanosoma Cruzi: Analysis Using Cell Electrophoresis, Lectins and Ultrastructural Cytochemistry. J. Submicrosc. Cytol. 18, 701–709. PubMed Abstract | Google Scholar 3097334

[B148] Stebel M. Vatta P. Ruaro M. E. del Sal G. Parton R. G. Schneider C. (2000). The growth suppressing gas 1 product is a GPI-Linked Protein. FEBS Lett. 481, 152–158. 10.1016/S0014-5793(00)02004-4 PubMed Abstract | 10.1016/S0014-5793(00)02004-4 | Google Scholar 10996315

[B149] Stone D. M. Hynes M. Armanini M. Swanson T. A. Gu Q. Johnson R. L. (1996). The Tumour-Suppressor Gene Patched Encodes a Candidate Receptor for Sonic Hedgehog. Nature 384, 129–134. 10.1038/384129a0 PubMed Abstract | 10.1038/384129a0 | Google Scholar 8906787

[B150] Su V. F. Jones K. A. Brodsky M. The I. (2007). Quantitative Analysis of Hedgehog Gradient Formation Using an Inducible Expression System. BMC Dev. Biol. 7, 43. 10.1186/1471-213X-7-43 PubMed Abstract | 10.1186/1471-213X-7-43 | Google Scholar 17484784 PMC1885436

[B151] Taipale J. Cooper M. K. Maiti T. Beachy P. A. (2002). Patched Acts Catalytically to Suppress the Activity of Smoothened. Nature 418, 892–896. 10.1038/nature00989 PubMed Abstract | 10.1038/nature00989 | Google Scholar 12192414

[B152] Tanaka Y. Okada Y. Hirokawa N. (2005). FGF-induced Vesicular Release of Sonic Hedgehog and Retinoic Acid in Leftward Nodal Flow Is Critical for Left-Right Determination. Nature 435, 172–177. 10.1038/nature03494 PubMed Abstract | 10.1038/nature03494 | Google Scholar 15889083

[B153] Tetley L. (1986). Freeze-fracture Studies on the Surface Membranes of Pleomorphic Bloodstream and *In Vitro* Transformed Procyclic Trypanosoma Brucei. Acta Trop. 43, 307–317. PubMed Abstract | Google Scholar 2882658

[B154] Toshimori K. Higashi R. Ōura C. (1985). Distribution of Intramembranous Particles and Filipin-Sterol Complexes in Mouse Sperm Membranes: Polyene Antibiotic Filipin Treatment. Am. J. Anat. 174, 455–470. 10.1002/aja.1001740408 PubMed Abstract | 10.1002/aja.1001740408 | Google Scholar 4083260

[B155] Truong M. E. Bilekova S. Choksi S. P. Li W. Bugaj L. J. Xu K. (2021). Vertebrate Cells Differentially Interpret Ciliary and Extraciliary cAMP. Cell 184, 2911–2926. e18. 10.1016/j.cell.2021.04.002 PubMed Abstract | 10.1016/j.cell.2021.04.002 | Google Scholar 33932338 PMC8450001

[B156] Tseng T. T. Gratwick K. S. Kollman J. Park D. Nies D. H. Goffeau A. (1999). The RND Permease Superfamily: an Ancient, Ubiquitous and Diverse Family that Includes Human Disease and Development Proteins. J. Mol. Microbiol. Biotechnol. 1, 107–125. PubMed Abstract | Google Scholar 10941792

[B157] Tukachinsky H. Kuzmickas R. P. Jao C. Y. Liu J. Salic A. (2012). Dispatched and Scube Mediate the Efficient Secretion of the Cholesterol-Modified Hedgehog Ligand. Cell Rep. 2, 308–320. 10.1016/j.celrep.2012.07.010 PubMed Abstract | 10.1016/j.celrep.2012.07.010 | Google Scholar 22902404 PMC3682496

[B158] Tyler K. M. Fridberg A. Toriello K. M. Olson C. L. Cieslak J. A. Hazlett T. L. (2009). Flagellar Membrane Localization via Association with Lipid Rafts. J. Cell Sci. 122, 859–866. 10.1242/jcs.037721 PubMed Abstract | 10.1242/jcs.037721 | Google Scholar 19240119 PMC2714428

[B159] van Meer G. Voelker D. R. Feigenson G. W. (2008). Membrane Lipids: where They Are and How They Behave. Nat. Rev. Mol. Cell Biol. 9, 112–124. 10.1038/nrm2330 PubMed Abstract | 10.1038/nrm2330 | Google Scholar 18216768 PMC2642958

[B160] Vyas N. Walvekar A. Tate D. Lakshmanan V. Bansal D. Cicero A. L. (2014). Vertebrate Hedgehog Is Secreted on Two Types of Extracellular Vesicles with Different Signaling Properties. Sci. Rep. 4. 10.1038/srep07357 PubMed Abstract | 10.1038/srep07357 | Google Scholar PMC4258658 25483805

[B161] Wang Q. Asarnow D. E. Ding K. Mann R. K. Hatakeyama J. Zhang Y. (2021). Dispatched Uses Na+ Flux to Power Release of Lipid-Modified Hedgehog. Nature 599, 320–324. 10.1038/s41586-021-03996-0 PubMed Abstract | 10.1038/s41586-021-03996-0 | Google Scholar 34707294 PMC8785653

[B162] Warner J. F. McCarthy A. M. Morris R. L. McClay D. R. (2014). Hedgehog Signaling Requires Motile Cilia in the Sea Urchin. Mol. Biol. Evol. 31, 18–22. 10.1093/MOLBEV/MST176 PubMed Abstract | 10.1093/MOLBEV/MST176 | Google Scholar 24124205 PMC3879447

[B163] Wassif C. A. Maslen C. Kachilele-Linjewile S. Lin D. Linck L. M. Connor W. E. (1998). Mutations in the Human Sterol Δ7-Reductase Gene at 11q12-13 Cause Smith-Lemli-Opitz Syndrome. Am. J. Hum. Genet. 63, 55–62. 10.1086/301936 PubMed Abstract | 10.1086/301936 | Google Scholar 9634533 PMC1377256

[B164] Wen X. Lai C. K. Evangelista M. Hongo J.-A. de Sauvage F. J. Scales S. J. (2010). Kinetics of Hedgehog-dependent Full-Length Gli3 Accumulation in Primary Cilia and Subsequent Degradation. Mol. Cell Biol. 30, 1910–1922. 10.1128/MCB.01089-09 PubMed Abstract | 10.1128/MCB.01089-09 | Google Scholar 20154143 PMC2849461

[B165] Wheatley D. N. (1995). Primary Cilia in Normal and Pathological Tissues. Pathobiology 63, 222–238. 10.1159/000163955 PubMed Abstract | 10.1159/000163955 | Google Scholar 8866794

[B166] Wheatley D. Wang A. M. Strugnell G. E. (1996). Expression of Primary Cilia in Mammalian Cells. Cell Biol. Int. 20, 73–81. 10.1006/cbir.1996.0011 PubMed Abstract | 10.1006/cbir.1996.0011 | Google Scholar 8936410

[B167] Wierbowski B. M. Petrov K. Aravena L. Gu G. Xu Y. Salic A. (2020). Hedgehog Pathway Activation Requires Coreceptor-Catalyzed, Lipid-dependent Relay of the Sonic Hedgehog Ligand. Dev. Cell 55, 450–467. e8. 10.1016/j.devcel.2020.09.017 PubMed Abstract | 10.1016/j.devcel.2020.09.017 | Google Scholar 33038332 PMC7686162

[B168] Willert K. Brown J. D. Danenberg E. Duncan A. W. Weissman I. L. Reya T. (2003). Wnt Proteins Are Lipid-Modified and Can Act as Stem Cell Growth Factors. Nature 423, 448–452. 10.1038/nature01611 PubMed Abstract | 10.1038/nature01611 | Google Scholar 12717451

[B169] Willnow T. E. Hilpert J. Armstrong S. A. Rohlmann A. Hammer R. E. Burns D. K. (1996). Defective Forebrain Development in Mice Lacking Gp330/megalin. Proc. Natl. Acad. Sci. U.S.A. 93, 8460–8464. 10.1073/pnas.93.16.8460 PubMed Abstract | 10.1073/pnas.93.16.8460 | Google Scholar 8710893 PMC38693

[B170] Wills R. C. Goulden B. D. Hammond G. R. V. (2018). Genetically Encoded Lipid Biosensors. MBoC 29, 1526–1532. 10.1091/mbc.E17-12-0738 PubMed Abstract | 10.1091/mbc.E17-12-0738 | Google Scholar 29953345 PMC6080648

[B171] Wong L. H. Levine T. P. (2017). Tubular Lipid Binding Proteins (TULIPs) Growing Everywhere. Biochimica Biophysica Acta (BBA) - Mol. Cell Res. 1864, 1439–1449. 10.1016/j.bbamcr.2017.05.019 PubMed Abstract | 10.1016/j.bbamcr.2017.05.019 | Google Scholar PMC5507252 28554774

[B172] Woods I. G. Talbot W. S. (2005). The You Gene Encodes an EGF-CUB Protein Essential for Hedgehog Signaling in Zebrafish. PLoS Biol. 3, e66. 10.1371/journal.pbio.0030066 PubMed Abstract | 10.1371/journal.pbio.0030066 | Google Scholar 15660164 PMC544551

[B173] Xiao X. Tang J.-J. Peng C. Wang Y. Fu L. Qiu Z.-P. (2017). Cholesterol Modification of Smoothened Is Required for Hedgehog Signaling. Mol. Cell 66, 154–162. e10. 10.1016/j.molcel.2017.02.015 PubMed Abstract | 10.1016/j.molcel.2017.02.015 | Google Scholar 28344083

[B174] Yao S. Lum L. Beachy P. (2006). The Ihog Cell-Surface Proteins Bind Hedgehog and Mediate Pathway Activation. Cell 125, 343–357. 10.1016/j.cell.2006.02.040 PubMed Abstract | 10.1016/j.cell.2006.02.040 | Google Scholar 16630821

[B175] Yen H.-Y. Hoi K. K. Liko I. Hedger G. Horrell M. R. Song W. (20182018). PtdIns(4,5)P2 Stabilizes Active States of GPCRs and Enhances Selectivity of G-Protein Coupling. Nature 559, 423–427. 10.1038/s41586-018-0325-6 PubMed Abstract | 10.1038/s41586-018-0325-6 | Google Scholar PMC6059376 29995853

[B176] Zeng X. Goetz J. A. Suber L. M. Scott W. J. Schreiner C. M. Robbins D. J. (2001). A Freely Diffusible Form of Sonic Hedgehog Mediates Long-Range Signalling. Nature 411, 716–720. 10.1038/35079648 PubMed Abstract | 10.1038/35079648 | Google Scholar 11395778

[B177] Zhang W. Kang J.-S. Cole F. Yi M.-J. Krauss R. S. (2006). Cdo Functions at Multiple Points in the Sonic Hedgehog Pathway, and Cdo-Deficient Mice Accurately Model Human Holoprosencephaly. Dev. Cell 10, 657–665. 10.1016/j.devcel.2006.04.005 PubMed Abstract | 10.1016/j.devcel.2006.04.005 | Google Scholar 16647303

[B178] Zhang Y. Bulkley D. P. Xin Y. Roberts K. J. Asarnow D. E. Sharma A. (2018). Structural Basis for Cholesterol Transport-like Activity of the Hedgehog Receptor Patched. Cell 175, 1352–1364. e14. 10.1016/j.cell.2018.10.026 PubMed Abstract | 10.1016/j.cell.2018.10.026 | Google Scholar 30415841 PMC6326742

[B179] Zhu A. J. Zheng L. Suyama K. Scott M. P. (2003). Altered Localization of Drosophila Smoothened Protein Activates Hedgehog Signal Transduction. Genes Dev. 17, 1240–1252. 10.1101/GAD.1080803 PubMed Abstract | 10.1101/GAD.1080803 | Google Scholar 12730121 PMC196058

